# Proxy indicators to support independent ageing-in-place in older people with frailty: a Delphi-style expert consultation survey

**DOI:** 10.1186/s12877-026-07968-9

**Published:** 2026-07-28

**Authors:** Schenelle Dayna Dlima, Abodunrin Quadri Aminu, Alex Hall, Chris Todd, Emma RLC Vardy

**Affiliations:** 1 School of Health Sciences, Faculty of Biology, Medicine and Health, The University of Manchester, Manchester, UK; 2 National Institute for Health and Care Research, Applied Research Collaboration - Greater Manchester, School of Health Sciences, Faculty of Biology, Medicine and Health, The University of Manchester, Manchester, UK; 3 National Institute for Health and Care Research Policy Research Unit in Healthy Ageing, School of Health Sciences, Faculty of Biology, Medicine and Health, The University of Manchester, Manchester, UK; 4 Manchester Academic Health Science Centre, Manchester, UK; 5 Manchester University NHS Foundation Trust, Manchester, UK; 6 Royal Oldham Hospital, Northern Care Alliance NHS Foundation Trust, Rochdale Road, Oldham, UK

**Keywords:** Ageing in place, Frailty, Independent living, Older people, Indicator, Delphi method

## Abstract

**Introduction:**

Frailty is a clinical syndrome associated with greater risk of functional limitations and dependency. However, older people with or at risk of frailty who live at home can still lead fulfilling lives, engage with their community, and maintain a good quality of life, that is, *age in place* well. To support this and prevent resource-intensive care use, this expert consultation survey aimed to identify proxy indicators that capture ageing-in-place experiences in community-dwelling older people with frailty.

**Methods:**

Using a modified Delphi approach, two survey rounds were conducted to build consensus on proxy ageing-in-place indicators in relation to frailty. Eighty-nine indicators were extracted from a rapid literature review and grouped into five themes: *Personal Characteristics of Older People*, *Place*, *Social Networks*, *Support*, and *Technology*. Eighteen experts in geriatric care and gerontology rated indicators for importance for ageing-in-place and feasibility of routine measurement. Consensus for importance and feasibility was defined as mean scores ≥ 4.0 (scale 1–5), suggesting indicators were extremely/very important for ageing-in-place *and* extremely/very feasible to measure routinely. Public contributors, who were experts by lived experiences of frailty and ageing-in-place, also reviewed the indicators and survey findings.

**Results:**

Only four proxy indicators reached consensus for importance *and* feasibility: “physical performance and mobility”, “multimorbidity”, “sensory function”, and “pressure ulcers” (all *Personal Characteristics*). Twenty-seven indicators were extremely/very important for ageing-in-place but not as feasible to measure routinely (17 related to *Personal Characteristics*, 4 to *Social Networks*, and 3 to *Place* and *Support*). Only five indicators (the four consensus indicators and “polypharmacy”) were extremely/very feasible to measure routinely. No technology-related indicators were extremely/very important or feasible to measure routinely. Mean scores of few indicators differed by experts’ occupations and areas of expertise. Public contributors generally agreed with the survey results but highlighted the importance of technology-related indicators (particularly “digital literacy”) and more integrated care records to support targeted care.

**Conclusions:**

This study lays the foundation for developing standardised proxy ageing-in-place indicators to monitor how older people with or at risk of frailty are living in their homes and communities. Future directions include assessing feasibility of capturing non-clinical indicators routinely; applying these indicators to guide personalised targeted interventions; and leveraging technology-related indicators to improve digital inclusion and literacy in older people to support ageing-in-place.

**Supplementary Information:**

The online version contains supplementary material available at https://doi.org/10.1186/s12877-026-07968-9.

## Introduction

Frailty is characterised by progressive declines in physiological reserve and functioning across multiple organ systems, compromising a person’s ability to cope with acute stressors and thereby accelerating their functional decline [1]. Although there is no universally accepted definition for frailty, the two most common approaches to conceptualise frailty are Fried’s phenotypic model (where frailty is a physical syndrome that clinically presents as five signs/symptoms) [2], and Rockwood’s cumulative deficit model (where frailty is a multidimensional risk state characterised by the accrual of deficits across multiple health domains) [3]. The progressive nature of frailty limits the ability to perform basic and instrumental activities of daily living (ADLs) over time [4–6], increasing the risk of falls, unplanned hospitalisations, worsening mobility, and transitions to care, nursing, and residential homes [7–10]. However, frailty is a dynamic condition and not a uni-directional path to permanent morbidity and dependency [11]. Identifying early stages of frailty and timely intervention can help prevent frailty from worsening, even improving chances of reversal [12, 13]. Moreover, in the context of age-related conditions such as frailty, there is a call to reframe the goal from “managing the condition” to “living well with the condition”. This means community-dwelling older people with frailty can still lead fulfilling lives, maintain a good quality of life, and age successfully with appropriate and tailored support, i.e. *age in place*, despite their functional and mobility limitations [14].

Ageing-in-place has gained increasing traction in policy and gerontology in the last 30 years [15], driven by the preference of older people to continue living in their own homes and communities, and the policy focus on reducing hospital use and institutionalisation [16]. Although there is no standard definition, ageing-in-place can be broadly defined as older people living in their own homes and community safely, independently, and comfortably for as long as possible, regardless of age, income, or ability level, without transitioning to long-term care facilities [17, 18]. It is important to acknowledge that ageing-in-place transcends the physical location, such as home and neighbourhood, older people reside in — the concept encompasses other domains including the built environment, community and social engagement, access to and receipt of support, older people’s perceptions, ability to maintain independence and make decisions, and general health and well-being [19].

Frailty is a complex condition with multiple contributing factors and heterogenous manifestations [20, 21]. Ageing-in-place experiences in older people are also heterogenous and influenced by several factors such as health status, cultural aspects, sociodemographic characteristics, home ownership status, neighbourhood characteristics, living status, financial stability, and access to formal support [22]. Thus, there are opportunities to address the multidisciplinary care needs of community-dwelling older people with frailty with policies and services that promote successful and independent ageing-in-place. These services and policies should encompass the multiple domains of ageing-in-place. This is where the goals for frailty management and ageing-in-place intersect — ageing-in-place policies should enable older people with frailty lead healthy and active lifestyles, sustain suitable economic activities, and live independently in their homes and communities for as long as possible, thereby lowering the risk of frailty exacerbation and long-term care transition [23].

Frailty has become a priority area in national health and policy agendas in the UK [16, 24–26], given the region’s ageing population trends and frailty’s considerable care costs [27]. There is also increasing focus on place-based care for frailty, which involves providing proactive, integrated health and social care closer to people’s homes and communities [26, 28]. Hence, delivering such place-based models of care for community-dwelling older people with frailty requires reliable and valid measures that capture the various ageing-in-place domains. There are several tools to assess frailty, but they mainly capture physical manifestations, functional limitations, and comorbidities. Some tools, such as the electronic Frailty Index (eFI) and Tilburg Frailty Indicator [29, 30], integrate domains such as psychological deficits and social vulnerability, signaling a shift from considering frailty as a merely biomedical disease to a more multifaceted condition. In the UK, the eFI comprises 36 deficits across clinical and functional domains [29], and its use is mandated in routine primary care practice to identify frailty for risk stratification [31]. However, there is limited research synthesising indicators that intersect the various ageing-in-place domains in the context of frailty, and on how items in frailty tools such as the eFI capture ageing-in-place experiences in the older people.

Considering this lack of standardised indicators for ageing-in-place, this study aimed to identify proxy indicators of ageing-in-place in community-dwelling older people with frailty. Given the absence of a validated composite ageing-in-place measure, these proxy indicators are indirect markers relating to an older person living in their chosen home (i.e. not in an assisted living environment or institutional setting) who may or may not be accessing services (such as homecare services) that enable them to remain in their homes and familiar communities for as long as possible. The proxy indicators include both subjective (self-reported) and objective (observed or clinically derived) measures. Hereafter, the terms “proxy indicators” and “indicators” will be used synonymously.

## Methods

**Fig. 1:**
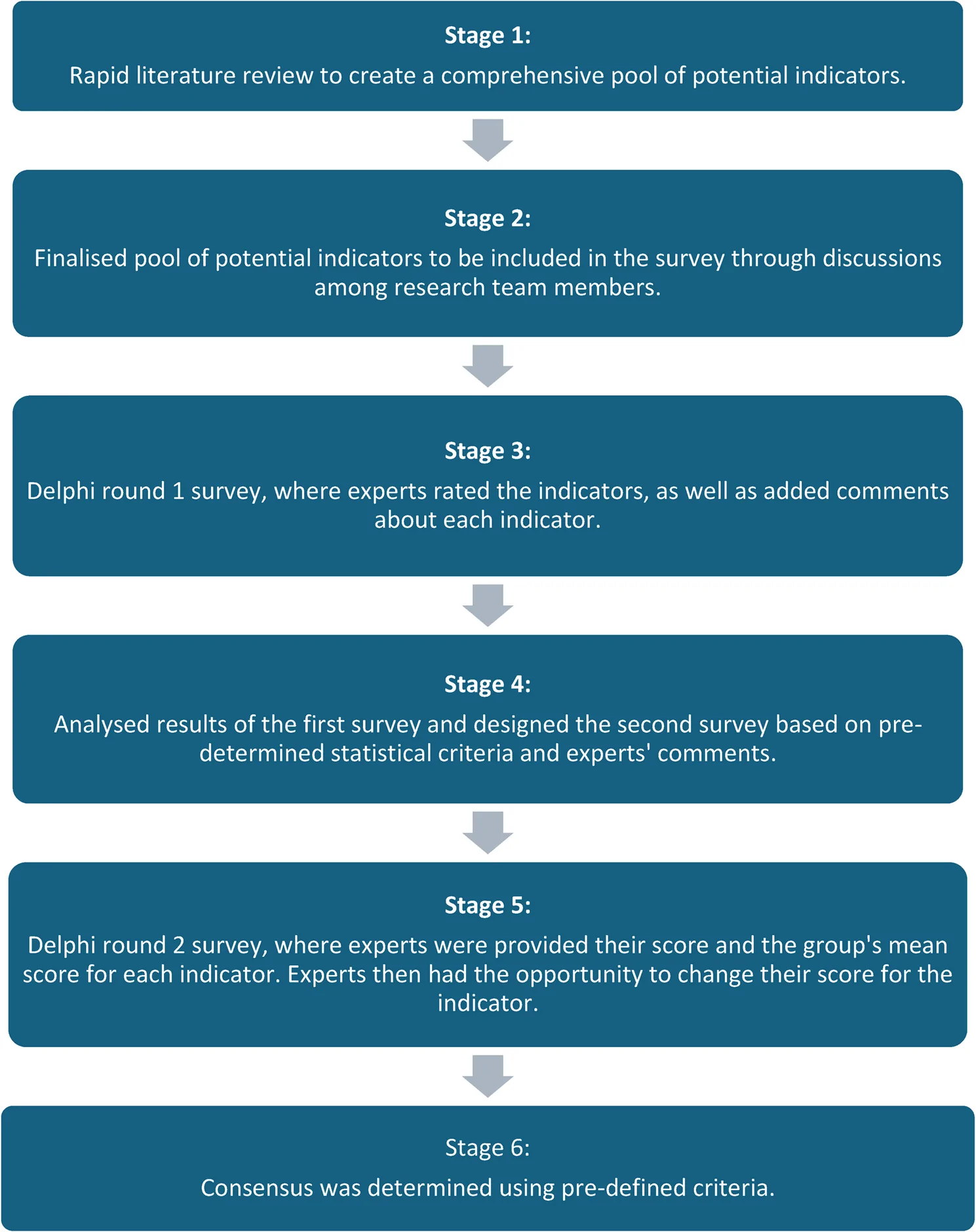
Stages of the Delphi-style expert consultation survey

This multi-stage, sequential study involved a rapid literature review to develop a comprehensive list of potential indicators, followed by a Delphi-style expert consultation survey. The methods used in this study were guided by the Conducting and REporting of DElphi Studies (CREDES) [32]. Fig. 1 shows the flowchart of the study process. The study was approved by the Proportionate University Research Ethics Committee of the University of Manchester (2024-18943-36066).

The Delphi methodology aims to evaluate consensus among professional and experience-based experts using multiple rounds of questioning [33, 34] and has been widely used in fields of life sciences and medicine [35]. The Delphi technique was selected to answer this study’s research questions because of its ability to generate insights on topics with limited information [35]. Given that research into quantifying healthy ageing in older people is still in its infancy, let alone research on quantifying ageing-in-place in the context of frailty, the Delphi technique would help build foundational knowledge in this area through consultations with multiple stakeholders.

### Expert eligibility criteria and recruitment

Experts with experience in gerontology, geriatric care, healthy ageing research, social and community care, and the care of older people with frailty were recruited. These were health and social care practitioners, researchers, and policy professionals. The co-authors’ professional networks were leveraged to recruit experts via convenience sampling. Email invitations were sent individually to potential experts deemed suitable for participation in the survey, based on their professional background and research expertise. Calls for experts were disseminated via mailing lists of relevant university research groups. These research groups were specific to healthy ageing and urban ageing. Potential experts were also invited through networking at a scientific conference. To recruit practitioners, the authors approached a community care centre that provides integrated care for older people with frailty in the city of Hull, England. Email invitations were sent to practitioners working in the centre, which included clinicians and social care staff.

The main inclusion criterion was the ability to participate in both rounds of the survey. If experts only completed the first survey, their response data were excluded from the final analysis.

### Stages 1–2: identifying candidate indicators for ageing-in-place in older people with frailty

A rapid literature review was conducted to create a comprehensive list of potential ageing-in-place indicators in the context of frailty. Following the guidance of the Cochrane Rapid Reviews Methods Group [36], Medline (Ovid), Embase, PsycINFO (one specialised database), and Cochrane Trials were used for the searches. The search terms were also narrow to ensure the most relevant literature was identified. The following concepts were included in the search strategy:

- (terms related to ageing-in-place)
- AND
- (indicator* OR measure* OR proxy OR candidate) OR (terms related to frailty)

Search terms for each database and the publication selection process are presented in Supplement 1. Data collection methods and outcomes assessed in these records were particularly examined to compile the list of ageing-in-place indicators in the context of frailty. In studies that used scales and questionnaires to collect data, items comprising these scales and questionnaires were closely looked at to identify additional indicators.

Potential indicators extracted from the search results and citation searches were grouped under the following ageing-in-place themes developed by Pani-Harreman et al. [19]:

- Personal characteristics (41 indicators): Sociodemographic, clinical, behavioural, and interpersonal characteristics of older people with or at risk of frailty.
- Place (18 indicators): Spatial context inside and around an older person’s home, where they interact with the physical, psychological, social, and geographic environment.
- Social networks (8 indicators): Frequency and nature of older people’s social interactions.
- Support (8 indicators): Formal support (from professionals and service providers) and informal support (from friends, family, neighbours, etc.).
- Technology (14 indicators): How older people perceive and use technologies, including those used for mobility support, information and communication technology (ICT), biotechnology, and ambient intelligence. These indicators largely mapped to the Technology Acceptance Model, which posits that acceptance of and intent to use technology are mediated by perceived usefulness and ease of use [37].

These themes were selected as they capture the multiple domains of ageing-in-place, enabling us to identify indicators most pertinent to older people with frailty. Both subjective indicators (quality of life, self-perceived health, neighbourhood aesthetics, etc.) and objective indicators (BMI, nutritional status, polypharmacy, etc.) were extracted to assess how feasibly information related to these indicators can be captured routinely in practice, both actively (such as self-reports) and passively (existing records). After compiling the comprehensive pool of potential indicators, the co-authors, who have different research and clinical expertise, trimmed the pool further through discussions. The complete indicator list and descriptions as presented in the survey are included in Supplement 2.

### Stages 3–5: Two-stage Delphi-style survey

After the potential indicators were finalised, a survey questionnaire was designed using Qualtrics (Supplement 3). The first survey asked experts to rate the ageing-in-place indicators on a scale of 1–5 (*not at all*, *somewhat*, *quite*, *very*, *extremely*) according to two criteria:

- Importance: how important is this indicator in understanding how older people with or at risk of frailty are ageing in place?
- Feasibility: how feasibly can this indicator be measured routinely in health and social care practice?

Experts could also add free text comments under each indicator. After round 1 was complete, mean scores and standard deviations (SDs) for importance and feasibility of each indicator were calculated and conventional content analysis was used to analyse the free-text responses. This has been done in previous modified Delphi studies [38, 39] where respondents assigned scores against multiple criteria. The survey was piloted by two practitioners (a general practitioner [GP] and dietitian) with research experience in frailty and healthy ageing. They provided feedback on the survey design, indicator list, clarity of the indicator descriptions, and participant information sheet.

The second survey was a bespoke survey for each expert, according to how they scored the indicators in round 1. This survey only included indicators where there was a difference between the expert’s assigned importance or feasibility score and the group’s overall mean importance or feasibility score (criteria for inclusion are provided in Table 1). Thus, “controlled feedback” was an aspect of this round, allowing experts to change their score depending on the results of the first survey. Summaries of the free-text responses from the first survey were also provided under relevant indicators. Both surveys were conducted between October 2024 and January 2025.

**Table 1:** Criteria for inclusion of indicators in the bespoke second surveys distributed to the experts

Group’s mean importance/feasibility score in the first survey (out of a possible score of 5)	Expert’s importance/feasibility score in the first survey (out of a possible score of 5)	Interpretation
< 4.0	≥ 4.0	Group did not consider this indicator *extremely/very* important or feasible. Expert rated this indicator as *extremely/very* important or feasible.
≥ 4.0 or < 2.0	2–3	Group considered this indicator either *not at all*,* very*, or* extremely* important or feasible. Expert rated this indicator as *somewhat/quite* important or feasible.
> 1.0	1.0	Group considered this indicator *at least somewhat* important or feasible. Expert considered this indicator *not at all* important or feasible.

### Stage 6: Analysing the results to identify indicators that reached consensus

Consensus for importance and feasibility was defined as mean importance and feasibility scores of ≥ 4.0 in the second survey. This means that the indicator was *extremely/very* important for an older person with frailty or at risk of frailty who is ageing in place, and *extremely/very* feasible to measure routinely in health and social care practice. To further examine how overall agreement in scores changed across both surveys, Kendall’s coefficients of concordance were calculated [40, 41].

Number of experts who assigned importance and feasibility scores of ≥ 4.0 for each indicator were examined Sub-group analyses were conducted to investigate how importance and feasibility scores for each indicator differed by occupation and areas of expertise using the non-parametric Kruskal–Wallis test. Statistical significance was set at a p-value of < 0.05. We also identified indicators that met only one criterion for consensus, i.e. only extremely/very important (mean score ≥ 4.0) but *not* extremely/very feasible to routinely measure (mean score < 4.0), and vice versa. All data analyses were conducted in Microsoft Excel and R statistical programme.

### Public and patient involvement

One-on-one discussions with five public contributors were conducted to seek their opinions on the survey results, potential missing indicators, ageing-in-place indicators that they considered important, and future directions for research and practice. “Public contributor” is an umbrella term that describes members of the public who work with researchers and health and care professionals during different stages of the research cycle. Recognising that older people and carers are also experts by virtue of their lived experiences of frailty and ageing-in-place, their inputs were considered valuable in understanding ageing-in-place promoters and barriers, and informing future steps for research, practice, and policy. However, as the survey asked experts to rate ageing-in-place indicators for feasibility of measurement in routine care, only practitioners and researchers were invited to complete the survey as they could provide more experience-based nuances on routinely capturing ageing-in-place indicators. Hence, public contributors were involved after survey completion. Public contributors were recruited via the National Institute for Health and Care Research (NIHR) Applied Research Collaboration network of public contributors. A summary of the study and expectations as contributors were advertised via a mailing list, and public contributors emailed the first author directly to express their interest in participating. The public contributors in this study had diverse lived experiences of frailty and ageing-in-place. They could therefore draw from personal knowledge and insights gained through either from their own experiences of living with frailty or through caring for someone with frailty. All public contributors lived in their own homes, enabling them to offer perspectives on managing frailty at home and ageing in place. A summary of the survey results (Supplement 4) and the complete indicator list were sent before the discussion for their review.

## Results

### Expert characteristics

Twenty experts completed the first survey, while 18 completed both rounds. Table 2 presents the characteristics of these 18 experts. Most experts worked in the Northwest and Yorkshire and The Humber regions of England (*n* = 12) and had 5–15 years of research/practice experience (*n* = 10). They were academics and researchers (*n* = 11), healthcare practitioners (*n* = 5), and social care and policy professionals (*n* = 2). Most experts stated that their area of expertise was community, hospital, and social care (*n* = 11). Their research areas included environmental epidemiology, applied research, blood pressure, falls prevention, digital equity, and healthy ageing.

**Table 2:** Sociodemographic and professional characteristics of experts who completed both surveys (*N* = 18)

Characteristic	*N*
Gender	
Men	2
Women	16
*Age group*	
25–34	6
35–44	5
45–54	5
55–64	1
65 and over	1
*Ethnicity*	
Asian / Asian British	4
Other ethnic group	1
White	13
*Location of work*	
Northwest England	7
Yorkshire and The Humber	5
London	3
East England	1
Northeast England	1
Southwest England	1
*Years of experience*	
Less than 5 years	5
5–15 years	10
16–25 years	2
25 years or more	1
*Profession*	
Academics and Researchers	11
Healthcare practitioners	5
Social care and Policy professionals	2
*Area of expertise*	
Community, hospital, and social care	11
Research and epidemiology	4
Health conditions and prevention	3
*Highest qualification*	
Bachelor’s degree	3
Master’s degree	5
Medical degree	2
Doctorate (PhD degree)	7
Other professional degree	1

### Survey results

#### Overall mean scores

The mean importance and feasibility scores for all 89 indicators after the two survey rounds are presented in Supplement 2. Ranges for the mean importance and feasibility scores were 2.5–4.8 and 2.1–4.4, respectively. “Physical performance and mobility” and “independence and confidence in undertaking activities of daily living” had the highest mean importance scores. “Multimorbidity” and “pressure ulcers” had the highest mean feasibility scores.

Only four indicators reached consensus, i.e. were considered extremely/very important for ageing-in-place in relation to frailty *and* extremely/very feasible to measure routinely. These were all *Personal Characteristics*: “physical performance and mobility”, “multimorbidity”, “sensory function”, and “pressure ulcers”. Twenty-seven indicators were considered extremely/very important for ageing-in-place but less feasible to measure routinely: 17 *Personal Characteristics*, 4 related to *Social Networks*, and 3 related to *Place* and *Support* each (Table 3). “Polypharmacy” was the only indicator considered extremely/very feasible to measure routinely, but not extremely/very important for ageing-in-place. All technology-related indicators were considered less important and less feasible to measure routinely (all mean scores < 4.0).

**Table 3:** Mean important and feasibility scores and standard deviations for indicators that were considered extremely/very important for ageing-in-place, and/or extremely/very feasibly to measure routinely (out of 18 experts)

Indicator	Category	Mean importance score (SD)	Mean feasibility score (SD)
*Extremely/very important and extremely/very feasible*			
Physical performance and mobility	Personal characteristics	4.8 (0.4)	4.3 (0.5)
Multimorbidity	Personal characteristics	4.4 (0.7)	4.4 (0.7)
Sensory function	Personal characteristics	4.7 (0.5)	4.2 (0.5)
Pressure ulcers	Personal characteristics	4.4 (0.6)	4.4 (0.6)
*Extremely/very important only*			
Independence and confidence in undertaking activities of daily living	Personal characteristics	4.8 (0.4)	3.7 (0.8)
Physical activity participation	Personal characteristics	4.7 (0.5)	3.6 (0.6)
Falls-related indicators	Personal characteristics	4.6 (0.5)	3.4 (0.6)
Memory and cognitive problems	Personal characteristics	4.6 (0.5)	3.3 (0.7)
Access to social support	Support	4.6 (0.6)	3.1 (0.7)
Lifestyle behaviours	Personal characteristics	4.4 (0.6)	3.3 (0.7)
Self-perceived health	Personal characteristics	4.3 (0.6)	3.6 (0.5)
Fatigue and tiredness	Personal characteristics	4.3 (0.6)	3.2 (0.7)
Social vulnerability	Personal characteristics	4.3 (0.5)	3.2 (0.7)
Access to public transport	Place	4.3 (0.6)	3.4 (1)
Home environment – occupational dimension^a^	Place	4.3 (0.6)	3 (0.8)
Social networks	Social networks	4.3 (0.6)	3.2 (0.6)
Food security	Support	4.3 (0.8)	3.1 (0.7)
Independence and confidence in undertaking instrumental activities of daily living	Personal characteristics	4.2 (1)	3.5 (0.7)
Quality of life	Personal characteristics	4.2 (0.5)	3.1 (0.8)
Loneliness	Personal characteristics	4.2 (0.6)	3.1 (0.5)
Nutritional status	Personal characteristics	4.2 (0.6)	3.1 (1)
Self-care ability	Personal characteristics	4.2 (0.4)	2.8 (0.9)
Psychological capacity	Personal characteristics	4.2 (0.5)	3.1 (0.8)
Land use mix – access ^b^	Place	4.2 (0.5)	3.1 (1.1)
Social participation – engagement	Social networks	4.2 (0.5)	2.8 (0.7)
Social participation – access	Social networks	4.2 (0.6)	2.9 (0.7)
Satisfaction with social relationships	Social networks	4.2 (0.5)	2.9 (0.6)
Satisfaction with social support	Support	4.2 (0.5)	2.7 (0.6)
Dyspnoea	Personal characteristics	4.1 (0.9)	3.2 (0.9)
Communication problems	Personal characteristics	4.1 (0.7)	2.8 (0.6)
Pain	Personal characteristics	4 (0.7)	3.1 (0.8)
*Extremely/very feasible only*			
Polypharmacy	Personal characteristics	3.6 (0.9)	4.3 (0.6)

*Abbreviations: SD* Standard deviation

^a^ “Home environment – occupational dimension” includes whether the home is easy to clean, people can easily move around in their home, or people can easily do the activities they need to and enjoy in their home

^b^ “Land use mix – access” refers to how easy a person finds accessing facilities such as recreational facilities, stores, banks, public transport, medical facilities, community and welfare services, and green spaces

Table 4 shows those indicators that at least 80% of experts considered extremely/very important for ageing-in-place in relation to frailty, and those considered extremely/very feasible to measure routinely (scores of 4 or 5). Regarding importance scores, 19 *Personal Characteristics*, 5 indicators related to *Place*, 4 related to *Social Networks*, and 3 related to *Support* were considered extremely/very important for ageing-in-place. Of these, eight *Personal Characteristics* received scores of 4 or 5 by all eighteen experts. “Physical performance and mobility” and “independence and confidence in undertaking activities of daily living” received the highest number of top ratings (score of 5), suggesting agreement on the importance of these indicators for ageing-in-place in relation to frailty. Regarding feasibility scores, only five *Personal Characteristics* were considered extremely/very feasible to routinely measure. All 18 experts agreed that “physical performance and mobility” was extremely/very feasible to routinely measure.

**Table 4:** Indicators that at least 80% of experts agreed were extremely/very important for ageing-in-place in relation to frailty, and extremely/very feasible to measure routinely

Indicator	Category	Mean score (SD)	Number of experts who assigned a score of 5	Number of experts who assigned a score of 4 or 5	Proportion of experts that assigned a score of 4 or 5 (%)
*Important for ageing-in-place*					
Physical activity participation	Personal characteristics	4.7 (0.5)	12	18	100
Physical performance and mobility	Personal characteristics	4.8 (0.4)	14	18	100
Independence and confidence in undertaking activities of daily living	Personal characteristics	4.8 (0.4)	14	18	100
Social vulnerability	Personal characteristics	4.3 (0.5)	6	18	100
Falls-related indicators	Personal characteristics	4.6 (0.5)	11	18	100
Self-care ability	Personal characteristics	4.2 (0.4)	4	18	100
Memory and cognitive problems	Personal characteristics	4.6 (0.5)	11	18	100
Sensory function	Personal characteristics	4.7 (0.5)	12	18	100
Self-perceived health	Personal characteristics	4.3 (0.6)	6	17	94
Fatigue and tiredness	Personal characteristics	4.3 (0.6)	7	17	94
Quality of life	Personal characteristics	4.2 (0.5)	5	17	94
Lifestyle behaviours	Personal characteristics	4.4 (0.6)	8	17	94
Psychological capacity	Personal characteristics	4.2 (0.5)	5	17	94
Pressure ulcers	Personal characteristics	4.4 (0.6)	8	17	94
Communication problems	Personal characteristics	4.1 (0.7)	4	17	94
Land use mix – access^a^	Place	4.2 (0.5)	5	17	94
Access to public transport	Place	4.3 (0.6)	7	17	94
Home environment – occupational dimension^b^	Place	4.3 (0.6)	6	17	94
Social participation	Social networks	4.2 (0.5)	4	17	94
Social networks	Social networks	4.3 (0.6)	7	17	94
Satisfaction with social relationships	Social networks	4.2 (0.5)	4	17	94
Food security	Support	4.3 (0.8)	7	17	94
Access to social support	Support	4.6 (0.6)	11	17	94
Satisfaction with social support	Support	4.2 (0.5)	5	17	94
Independence and confidence in undertaking instrumental activities of daily living	Personal characteristics	4.2 (1)	8	16	89
Loneliness	Personal characteristics	4.2 (0.6)	6	16	89
Nutritional status	Personal characteristics	4.2 (0.6)	5	16	89
Multimorbidity	Personal characteristics	4.4 (0.7)	9	16	89
Social participation	Social networks	4.2 (0.6)	5	16	89
Safety and security in community or neighbourhood	Place	3.8 (0.9)	2	15	83
Neighbourhood walkability	Place	3.8 (0.6)	1	15	83
*Feasible to measure routinely*					
Physical performance and mobility	Personal characteristics	4.3 (0.5)	6	18	100
Sensory function	Personal characteristics	4.2 (0.5)	5	17	94
Polypharmacy	Personal characteristics	4.3 (0.6)	7	17	94
Pressure ulcers	Personal characteristics	4.4 (0.6)	8	17	94
Multimorbidity	Personal characteristics	4.4 (0.7)	9	16	89

*Abbreviations: SD* Standard deviation

^a^ “Land use mix – access” refers to how easy a person finds accessing facilities such as recreational facilities, stores, banks, public transport, medical facilities, community and welfare services, and green spaces

^b^ “Home environment – occupational dimension” includes whether the home is easy to clean, people can easily move around in their home, or people can easily do the activities they need to and enjoy in their home

#### Mean scores by experts’ occupations and areas of expertise

Table 5 presents the indicators for which mean scores showed statistically significant differences across experts’ occupations. Healthcare practitioners generally assigned lower importance scores to indicators related to *Social Networks* and *Technology* compared with academics and researchers, and social care and policy professionals. No indicator had mean feasibility scores that showed statistically significant differences by experts’ occupations.

**Table 5:** Indicators for which mean scores showed statistically significant differences (*p* < 0.05) across experts’ occupations

Indicator	Category	Mean scores by experts’ occupations			
		Academics and researchers (*n* = 11)	Healthcare practitioners (*n* = 5)	Social care and policy professionals (*n* = 2)	*P*-values
*Important for ageing-in-place*					
Psychological capacity	Personal characteristics	4.3	3.8	5	0.02
Dizziness	Personal characteristics	3.8	2.8	4.5	0.03
Attachment to place	Place	3.6	3	4.5	0.04
Land use mix – access ^a^	Place	4.3	3.8	5	0.02
Safety and security in community or neighbourhood	Place	4.2	2.8	4	0.02
Home environment – physical dimension (structure, services, and facilities)	Place	3.8	3	4.5	0.04
Social participation – access	Social networks	4.5	3.6	4	0.03
Social participation – satisfaction	Social networks	3.8	2.8	4	0.004
Social networks	Social networks	4.5	3.8	5	0.03
Satisfaction with social relationships	Social networks	4.2	3.8	5	0.01
General satisfaction with technology	Technology	3.3	2.4	3	0.02
Perceived usefulness of technology	Technology	3.2	2.2	3	0.02
Reliability of the technology	Technology	3.2	2.4	3.5	0.02
Comfort with technology use	Technology	3.3	2.4	3.5	0.02
Subjective norms about technology	Technology	3.2	2.2	2.5	0.02

^a^ “Land use mix – access” refers to how easy a person finds accessing facilities such as recreational facilities, stores, banks, public transport, medical facilities, community and welfare services, and green spaces

Table 6 presents the indicators for which mean scores showed statistically significant differences across experts’ areas of expertise. Mean importance scores of three indicators and mean feasibility scores of seven indicators showed statistically significant differences by areas of expertise Experts in community, hospital, and social care generally assigned higher feasibility scores for *Personal Characteristics* compared with the other experts. These *Personal Characteristics* were mostly clinical in nature.

**Table 6:** Indicators for which mean scores showed statistically significant differences (*p* < 0.05) across experts’ areas of expertise

Indicator	Category	Mean scores by experts’ areas of expertise			
		Community, hospital, and social care (*n* = 11)	Research and epidemiology (*n* = 4)	Health conditions and prevention (*n* = 3)	*P*-values
*Important for ageing-in-place*					
Psychological capacity	Personal characteristics	4.1	4	5	0.02
Foot problems	Personal characteristics	2.8	3.5	4.3	0.02
Land use mix – access^a^	Place	4	4.2	5	0.02
*Feasible to measure routinely*					
Nutritional status	Personal characteristics	3.5	2.8	2	0.04
Multimorbidity	Personal characteristics	4.5	3.8	5	0.04
Polypharmacy	Personal characteristics	4.4	3.8	5	0.02
Dyspnoea	Personal characteristics	3.5	2.2	3.3	0.04
Pressure ulcers	Personal characteristics	4.5	3.8	5	0.03
Working capacity	Personal characteristics	2.4	2	1.3	0.03
Access to public transport	Place	3.3	2.8	4.7	0.04

^a^ “Land use mix – access” refers to how easy a person finds accessing facilities such as recreational facilities, stores, banks, public transport, medical facilities, community and welfare services, and green spaces

### Coordination of experts’ scores

Kendall’s coefficients of concordance were calculated to examine coordination of experts’ importance and feasibility scores (Table 7). When accounting scores of all indicators, the coefficient value increased from 0.338 in the first survey to 0.512 in the second survey, indicating greater consensus after two rounds. Coefficient value of 0.512 suggests moderate, positive coordination among experts’ scores.

**Table 7:** Significance test results to examine agreement among experts’ scores

	Kendall’s coefficient (W)	χ^2^	*P*-value
Round 1 survey	0.338	1076.9	**0.000**
Round 2 survey	0.512	1629.8	**0.000**
Round 2 importance scores	0.523	828.6	**0.000**
Round 2 feasibility scores	0.377	597.2	**0.000**

P-values in bold are statistically significant (*p* < 0.05)

Upon analysing the coefficients for the second-survey importance and feasibility scores separately, we found a greater degree of concordance in the importance scores than in the feasibility scores (0.523 vs. 0.377, respectively).

### Qualitative insights from experts

Summaries of the free-text responses provided by the experts for each indicator are provided in Supplement 2. Experts suggested that some ageing-in-place indicators may not be specific to frailty, such as food insecurity, pain, urinary tract symptoms, resilience, self-perceived stress, communication problems, polypharmacy, and dizziness. Some experts questioned the relevance of some indicators for both frailty and ageing-in-place, such as personality factors, working capacity, and the physical environment to accommodate technological interventions.

Regarding measuring the indicators, experts highlighted that some can be captured using wearable devices and digital tools (physical activity participation, sleep-related indicators, ambience and space of the home environment), self-reports (fatigue and tiredness, certain aspects of the home environment, social participation), and existing tests (Timed-Up-and-Go test for physical performance; questionnaires and scales for ADLs, social networks, or fear of falling). Experts also pointed out that some *Personal Characteristics* are already measured routinely in practice, such as BMI, nutritional status, polypharmacy (through medication reviews), and sensory function. Few indicators related to *Personal Characteristics* (lifestyle behaviours and multimorbidity) and *Place* (land use mix; residential density; street connectivity; public transport access; neighbourhood resources) can be accessed from electronic health records, records from the Office for National Statistics (ONS) and local authorities, and publicly available spatial datasets.

### Public contributors’ insights

Supplement 5 summarises the discussions with the public contributors, structured by pre-determined themes (i.e. directly asked during the discussions) and those that emerged during analysis. Mean age of the public contributors was 70.2 years at the time of the discussions (range: 58–79 years) and included three men and two women. Regarding their lived experiences of frailty and ageing-in-place, two contributors had a history of mobility limitations and sensory problems, and one was a full-time carer of a loved one living with multiple long-term conditions. Two contributors had previous experience in providing care in formal settings, including one who worked in social care for ten years.

All public contributors generally agreed with the experts’ scores on the four extremely/very important *and* feasible indicators. The discussions suggested that indicators related to social support; accessibility to neighbourhood resources; neighbourhood design; attachment to place; independence and confidence in ADLs; self-care ability; adaptability; resilience; and digital literacy are important for older people ageing in place. Public contributors acknowledged that technology-related indicators are important to consider, especially given the increased digitalisation of essential services. Digital literacy was explicitly highlighted by some public contributors, and they acknowledged gaps in digital inclusion among older people and the need for education in using technology and digital services. They also emphasised the lack of integration among the different services they use, rendering it challenging for service providers to understand the complete picture of a person’s health and living status. Regarding future directions, public contributors stated how routine records should be expanded to include non-clinical information related to *Place* and *Social Networks*, which should be integrated with existing records that can be accessed by all service providers.

## Discussion

This two-stage Delphi style survey involving 18 experts sought to identify proxy indicators to support independent ageing-in-place in older people with or at risk of frailty. Of 89 indicators, only four *Personal Characteristics* met the criteria for consensus, i.e. were considered extremely/very important for ageing-in-place in relation to frailty *and* extremely/very feasible to measure routinely: “physical performance and mobility”, “multimorbidity”, “sensory function”, and “pressure ulcers”. These indicators are likely to be assessed during routine healthcare interactions through self-reports, assessments, or observations, and existing clinical records can provide information about “multimorbidity”. Twenty-seven indicators were considered extremely/very important for ageing-in-place but not as feasible to measure routinely. Although these were predominantly *Personal Characteristics*, indicators related to public transport, the home environment (occupational dimension), proximity to amenities and services (land use mix), social networks, and social support received importance scores of ≥ 4.0, and their importance was emphasised during the discussions with public contributors. These results were consistent with a previous Delphi survey on important community-based factors for ageing-in-place [42], where carers and healthcare stakeholders agreed that a safe home environment and strong relationships with friends, family, and the community are important for older people ageing in place. In the context of frailty, association of these indicators with frailty, disability, and functional limitations have been established, with newer conceptualisations of frailty, such as social frailty, cognitive frailty, and psychological frailty, recognising non-clinical and non-physical risk factors [21, 43–51]. These recent frailty conceptualisations include ageing-in-place indicators proposed in this survey in their operationalisation, reiterating the importance and relevance of these indicators for frailty and ageing-in-place.

Experts did not consider technology-related indicators either as important for ageing-in-place in relation to frailty or feasible to measure routinely. Research suggests older people with frailty, sensory impairments, ADL limitations, and other comorbid conditions are less likely to accept and/or use ICT and remote monitoring technologies [52–55], which may explain the relatively lower importance scores assigned by experts. However, the public contributors and experts’ free-text responses highlighted the usefulness of technology in performing instrumental ADLs (such as online shopping) and staying in touch with loved ones. They also emphasised the need for some levels of digital literacy to navigate the increasing digitalisation of essential services. Indeed, a recent report by Age UK echoed these findings, highlighting how 4.7 million people aged ≥ 65 in the UK lack basic skills to use the internet successfully, increasing their frustration and disempowerment and limiting their access to essential services such as booking GP appointments, council services, and banking [56]. Given the growing recognition of technological solutions to facilitate ageing-in-place and frailty management [57, 58], these indicators can help determine *whether* and *which type* of technology is best suited to support an older person with or at risk of frailty who is ageing in place.

### Future research implications for ageing-in-place indicators in relation to frailty

This research adds to the growing literature on developing quantitative measures of healthy ageing and aspects of ageing-in-place in the context of frailty [59–67]. The survey provides real-world insights on how practitioners, researchers, and policy professionals consider the feasibility of measuring these indicators routinely; only four extremely/very important indicators were considered extremely/very feasible to routinely measure. Several short-form questionnaires exist to assess non-clinical indicators such as the Neighbourhood Environment Walkability Scale [68], Lubben Social Network Scale, and Senior Technology Acceptance Model questionnaire [69]. Future research should explore integrating such short-form questionnaires into routine practice and determining where in the care pathway these indicators can be best assessed, while accounting for burdens on already overstretched workforces and carers. Experts noted that some indicators related to *Place* are also available in national and regional administrative databases; thus, there are opportunities to leverage such data sources to understand ageing-in-place trends, especially given regional, neighbourhood-level, and socioeconomic disparities in frailty prevalence [70, 71]. For example, Yang et al. developed “Ageing in Place Classifications” for older people aged ≥ 50 using geodemographic data from various sources such as the UK Census, National Health Service (NHS) England data, and the British Population Survey [23]. Thus, future research can explore whether direct or proxy measures of these indicators are available in these databases, and whether these measures can be harmonised feasibly to examine how older people are ageing in place, and compare built and social environment characteristics across frailty “hotspots”.

Replicating this survey with a larger and more diverse pool of professional experts would help validate the importance and feasibility of these ageing-in-place indicators, or shed light on geographical, context-specific heterogeneities in the scores. In the context of ageing-in-place, research indicates mediating roles and complex interactions among these indicators [44, 48, 72]. Hence, these indicators can be used to conduct multi-level analyses to build a frailty-specific ageing-in-place framework that includes these interactions and pathways, helping address multi-disciplinary care needs.

### Practice and policy implications for ageing-in-place indicators in relation to frailty

This research is relevant in the current clinical and policy space around preventing the onset and progression of frailty and implementing ageing-in-place policies to support community-dwelling older people. In the UK, a supply–demand gap in adult social care (which includes care/nursing homes and homecare) persists, with only about 42% of requests resulting in some form of service funded by the state [73]. Demand for occupied places in care homes by older people is projected to rise by about 26,000 between 2024 and 2030 [74]. However, this was reported as a modest increase, largely due to policy commitments favouring homecare services and supported living in the community. Whilst this may indicate a positive shift towards promoting ageing-in-place, access to adult social care funded by local authorities is highly restricted, i.e. only those with the highest level of need qualify for state-funded care packages [75]. In addition, frailty is associated with significant health and social care use and costs [76, 77]. Thus, institutionalisation and need for new homecare services can incur high financial and resource costs to older people with or at risk of frailty, their carers, and the already strained social and welfare sector in the UK. This warrants indicators with standardised conceptual and operational definitions to understand how older people are ageing in place, helping identify at-risk individuals and neighbourhoods to deliver timely and appropriate intervention.

Regarding the relevance of these ageing-in-place indicators for intervention and care planning, previous research [42, 78–80], policy strategies [16, 24, 25, 28], and the discussions with the public contributors repeatedly emphasised two descriptors: “person-centred” and “integrated”. Both frailty and ageing-in-place are dynamic processes, and these multi-domain indicators can help predict and meet the evolving needs of older people with frailty living in their own homes and communities, i.e. personalising the care and support provided to them. Delivering appropriate person-centred care necessitates going beyond a person’s medical history to incorporate a deeper understanding of their physical and social environments. Interestingly, the electronic Frailty Index 2 (eFI2), which builds on the nationally implemented eFI in the UK, does not include two of the four clinically leaning, extremely/very important and feasible indicators as deficits (“sensory impairments” and “pressure ulcers”) that made up the original eFI [29, 81]. Moreover, “polypharmacy”, which was not considered extremely/very important by experts in this survey, was one of the highest-weighted deficits in the eFI2. However, unlike the original eFI, eFI2 now includes environment problems, lifestyle behaviours (smoking status and alcohol intake levels), and ADLs such as dressing and grooming and medication management as deficits, which experts considered extremely/very important in this survey. This signals a real-world recognition of the non-clinical contributors of frailty and adverse outcomes related to ageing-in-place, such as care home transitions and requirement for new homecare packages. These outcomes were assessed in the eFI2 study [81]. In their editorial of the eFI2, Romero-Ortuno and Keevil [82] highlighted how frailty management should take a more holistic approach (managing underlying conditions, nutrition, physical activity, health education, social support) rather than the overtly clinical approach currently in the NHS GP contract (falls referrals, deprescribing, advanced care planning). Thus, supplementing frailty identification using tools such as the eFI2 with ageing-in-place indicators can provide nuanced insights on an older person’s health status and quality of life, forming the basis of more tailored care and management strategies.

In the policy space, there is a call to better integrate health and social care services to facilitate joined-up and holistic care [16, 24, 25, 83]. However, this requires harmonised records and datasets, which are lacking in the UK due to the fragmented structure of primary care, secondary care, social care, voluntary, and council services. Moreover, data on older people who do not interact with services are almost non-existent, which may contribute to intervention delays for those in the potentially reversible stages of frailty. Building Integrated Care Systems (ICSs), involving “triple integration of primary and specialist care, physical and mental health services, and health with social care” [16, p. 29] has been reported as a priority area for targeted solutions that enable community-dwelling older people maximise their independence. These ICSs are local partnerships composed of area-specific NHS organisations and upper-tier local authorities, facilitating proactive and joined-up care in the community. A few are even targeted towards older people with frailty [84–86], highlighting the real-world potential of integrated care in addressing frailty in the community. Given how the relatively lower feasibility scores assigned by experts in this survey indicate challenges in measuring non-clinical indicators routinely, it may be worth exploring what information already available in ICSs can serve as proxies for these indicators. These data from ICSs should be integrated with those from administrative data sources, such as the ONS [25], to help disentangle the clinical, behavioural, environmental, and social determinants of older people’s ability to remain independent, age in place well, and continue doing what they value.

Regarding the technology-related indicators, their relevance is underscored in national policy documents, which recognise the potential of technology to transform different stages of a patient’s healthcare journey [16] and to help people with frailty remain independent and continue living at home [26]. Research suggests that older people have positive attitudes towards technology, are generally open to using technology for various purposes, and are open to sharing their information with healthcare practitioners if it meant it would support their care [87–89]. However, privacy concerns and digital exclusion among older people persist, which were echoed in the discussions with public contributors. This may limit older people’s use of essential services that are increasingly being digitalised and their access to newer technologies that could help them maintain their independence. The technology-related indicators could help understand how older people and practitioners perceive and use different kinds of technology, from the internet to smart home modifications. This could help determine suitability of technological solutions to manage frailty and support independent living in the community, and identify older people who would benefit from tailored training to bridge the digital divide.

### Strengths and limitations of this study

This survey included subjective and objective indicators, which are both important to provide a comprehensive view of successful ageing-in-place in relation to frailty [90]. It also assessed the feasibility of measuring subjective indicators routinely, with experts providing additional comments based on their practical experiences. This provided insights into the current use and potential applicability of tools such as self-reports, questionnaires, etc., in routine practice. It should be recognised that public contributors’ understanding of feasibility may have differed from that of the professional experts. For example, assessing “sensory function” may be feasible from a patient’s perspective (if it takes a few minutes), but not as much for a clinician with multiple patients to assess in a day. Nevertheless, the public contributors helped validate the importance of some indicators, thereby strengthening the findings. Results of the sub-group analyses of the mean scores should be interpreted with caution as the number of experts across the categories of occupations and areas of expertise was highly imbalanced. The survey also only included experts based in England, limiting generalisability of the indicators’ importance and feasibility to other geographies and contexts. However, the general trends can be compared with those in future surveys that include a more diverse pool of experts. Other limitations of the Delphi technique include time constraints among experts (which may have led to survey fatigue in the later stages), the “bandwagon effect” (where experts resort to aligning their scores with the group’s scores without adequate reflection), and biases based on occupation and areas of expertise [91]. Nevertheless, this survey technique built on the growing body of ageing-in-place literature by allowing experts draw from their professional experience to assess the 89 ageing-in-place indicators.

## Conclusions

This two-stage, Delphi-style survey asked experts based in England to rate ageing-in-place indicators based on importance in relation to frailty and feasibility of routine measurement. Only four of the 89 indicators met the pre-determined criteria for consensus for importance and feasibility. The survey highlighted how some important indicators were not considered as feasible to measure routinely, and technology-related indicators were considered neither important nor feasible to measure routinely. The findings have research, practice, and policy implications, given the growing focus on addressing frailty as a public health problem, supporting older people to remain independent in their homes and familiar communities, and preventing resource-intensive care use. Based on the survey results and discussions with public contributors, future directions include: (1) involving a more diverse pool of experts (by profession and lived experiences) in further surveys and qualitative research; (2) examining the feasibility of routinely collecting and harmonising data on the important ageing-in-place indicators; (3) exploring the potential of these multi-domain indicators to deliver targeted, holistic, and optimal care to older people with or at risk of frailty living in their homes; and (4) leveraging technology-related indicators to promote digital inclusion and literacy among older people, which could shape their perceptions on technological solutions for independent ageing-in-place.

## Supplementary Information

Supplement 1. Search strategy for rapid review to identify potential ageing-in-place indicators: Includes broad eligibility criteria for rapid review, search strategies, and flowchart of study selection process.

Supplement 2. Descriptions, mean scores, and experts’ comments related to the ageing-in-place indicators: An Excel sheet with the indicator name, description, category, mean importance and feasibility scores with standard deviations, and experts’ comments on the indicator.

Supplement 3. Survey questionnaire distributed to experts: Contents of the first survey as designed in Qualtrics.

Supplement 4. Lay summary report of the survey: Visual report summarising the survey results that was sent to the public contributors before the discussions.

Supplement 5. Thematic summary of the discussions with public contributors regarding their feedback and insights on the ageing-in-place indicators: Summary of the discussions with public contributors mapped to questions asked during the discussions or themes that emerged during the discussions.

## Data Availability

All data generated or analysed during this study are included in this published article and its supplementary additional files.

## Abbreviations

ADLs: Activities of daily living
BMI: Body mass index
eFI: Electronic Frailty Index
GP: General Practitioner
ICS: Integrated Care System
ICT: Information and communication technology
NHS: National Health Service
ONS: Office for National Statistics
SD: Standard deviation

## References

[1] Clegg A, Young J. The frailty syndrome. Clin Med. 2011;11(1):72–5. 10.7861/clinmedicine.11-1-72 PMC5873811 21404792

[2] Fried LP, Tangen CM, Walston J, Newman AB, Hirsch C, Gottdiener J, et al. Frailty in older adults: evidence for a phenotype. Journals Gerontology: Ser A. 2001;56(3):M146–57. 10.1093/gerona/56.3.m146 11253156

[3] Rockwood K, Mitnitski A. Frailty in relation to the accumulation of deficits. Journals Gerontology: Ser A. 2007;62(7):722–7. 10.1093/gerona/62.7.722 17634318

[4] Álvarez-Bustos A, Carnicero‐Carreño JA, Sanchez‐Sanchez JL, Garcia‐Garcia FJ, Alonso‐Bouzón C, Rodríguez‐Mañas L. Associations between frailty trajectories and frailty status and adverse outcomes in community‐dwelling older adults. J Cachexia Sarcopenia Muscle. 2022;13(1):230–9. 10.1002/jcsm.12888 PMC8818602 34951157

[5] Kaskirbayevaa D, West R, Jaafari H, King N, Howdon D, Shuweihdif F, et al. Progression of frailty as measured by a cumulative deficit index: a systematic review. Ageing Res Rev. 2023;94:101789. 10.1016/j.arr.2022.101789 36396032

[6] Sánchez-García S, García-Peña C, Salvà A, Sánchez-Arenas R, Granados-García V, Cuadros-Moreno J, et al. Frailty in community-dwelling older adults: association with adverse outcomes. Clin Interv Aging. 2017;12:1003–11. 10.2147/CIA.S139860 PMC5498785 28721028

[7] Bentur N, Sternberg SA, Shuldiner J. Frailty transitions in community-dwelling older people. Isr Med Association J. 2016;18(8):449–53. 28471574

[8] Cegri F, Orfila F, Abellana RM, Pastor-Valero M. The impact of frailty on admission to home care services and nursing homes: Eight-year follow-up of a community-dwelling, older adult, Spanish cohort. BMC Geriatr. 2020;20(1):281. 10.1186/s12877-020-01683-9 PMC7412800 32762773

[9] Chen R, Zhao WB, Zhang XP, Liang H, Song NN, Liu ZY, et al. Relationship between frailty and long-term care needs in Chinese community-dwelling older adults: a cross-sectional study. BMJ Open. 2022;12(4):e051801–e. 10.1136/bmjopen-2021-051801 PMC9058676 35487750

[10] Heppenstall CP, Wilkinson TJ, Hanger HC, Keeling S, Pearson J. Factors related to care home admission in the year following hospitalisation in frail older adults. Age Ageing. 2011;40(4):513–6. 10.1093/ageing/afr045 21622672

[11] Ofori-Asenso R, Lee Chin K, Mazidi M, Zomer E, Ilomaki J, Ademi Z, et al. Natural regression of frailty among community-dwelling older adults: a systematic review and meta-analysis. Gerontologist. 2020;60(4):e286–98. 10.1093/geront/gnz064 31115434

[12] Puts MTE, Toubasi S, Andrew MK, Ashe MC, Ploeg J, Atkinson E, et al. Interventions to prevent or reduce the level of frailty in community-dwelling older adults: a scoping review of the literature and international policies. Age Ageing. 2017;46(3):383–92. 10.1093/ageing/afw247 PMC5405756 28064173

[13] Travers J, Romero-Ortuno R, Bailey J, Cooney M-T. Delaying and reversing frailty: a systematic review of primary care interventions. Br J Gen Pract. 2019;69(678):e61. 10.3399/bjgp18X700241 PMC6301364 30510094

[14] Gichu M, Harwood RH. Measurement of healthy ageing. Age Ageing. 2023;52(Supplement4):iv3–5. 10.1093/ageing/afad118 PMC10615059 37902515

[15] Ahn M, Kang J, Kwon HJ. The Concept of Aging in Place as Intention. Gerontologist. 2020;60(1):50–9. 10.1093/geront/gny167 30605499

[16] NHS England. The NHS Long Term Plan. 2019. Available from: https://www.longtermplan.nhs.uk/wp-content/uploads/2019/08/nhs-long-term-plan-version-1.2.pdf. [cited 2025 22 July].

[17] CDC. Healthy Places Terminology. 2009. Available from: https://www.cdc.gov/healthyplaces/terminology.htm. [cited 2025 22 July].

[18] Grimmer K, Kay D, Foot J, Pastakia K. Consumer views about aging-in-place. Clin Interv Aging. 2015;10:1803–11. 10.2147/CIA.S90672 PMC4639513 26604723

[19] Pani-Harreman KE, Bours GJJW, Zander I, Kempen GIJM, van Duren JMA. Definitions, key themes and aspects of ‘ageing in place’: a scoping review. Aging Soc. 2021;41(9):2026–59.

[20] Wang X, Hu J, Wu D. Risk factors for frailty in older adults. Med (Baltim). 2022;101(34):e30169–e. 10.1097/MD.0000000000030169 PMC9410572 36042657

[21] Boucham M, Salhi A, El Hajji N, Gbenonsi GY, Belyamani L, Khalis M. Factors associated with frailty in older people: an umbrella review. BMC Geriatr. 2024;24(1):737–19. 10.1186/s12877-024-05288-4 PMC11376099 39237866

[22] Bosch-Farré C, Malagón-Aguilera MC, Ballester-Ferrando D, Bertran-Noguer C, Bonmatí-Tomàs A, Gelabert-Vilella S, et al. Healthy ageing in place: enablers and barriers from the perspective of the elderly. A qualitative study. Int J Environ Res Public Health. 2020;17(18):1–23. 10.3390/ijerph17186451 PMC7559318 32899744

[23] Yang Y, Dolega L, Darlington-Pollock F. Ageing in Place Classification: Creating a geodemographic classification for the ageing population in England. Appl Spatial Analysis. 2023;16:583–623. 10.1007/s12061-022-09490-y. PMC9742038 36532714

[24] British Geriatrics Society. Joining the dots: A blueprint for preventing and managing frailty in older people. 2023. Available from: https://www.bgs.org.uk/joining-the-dots-a-blueprint-for-preventing-and-managing-frailty-in-older-people-0. [cited 2025 July 26].

[25] Department of Health and Social Care. Chief Medical Officer’s annual report 2023: health in an ageing society. 2023. Available from: https://www.gov.uk/government/publications/chief-medical-officers-annual-report-2023-health-in-an-ageing-society. [updated 16 November 2023; cited 2025 26 July].

[26] UK Government. Fit for the Future: The 10 Year Health Plan for England. 2025. Available from: https://www.gov.uk/government/publications/10-year-health-plan-for-england-fit-for-the-future. [updated 30 July 2025; cited 2026 2 June].

[27] Fogg C, England T, Zhu S, Jones J, de Lusignan S, Fraser SDS, et al. Primary and secondary care service use and costs associated with frailty in an ageing population: longitudinal analysis of an English primary care cohort of adults aged 50 and over, 2006–2017. Age Ageing. 2024;53(2):afae010. 10.1093/ageing/afae010 PMC10857897 38337044

[28] NHS England. Neighbourhood health guidelines 2025/26. 2025. Available from: https://www.england.nhs.uk/long-read/neighbourhood-health-guidelines-2025-26/. [updated 25 March 2025; cited 2025 22 July].

[29] Clegg A, Bates C, Young J, Ryan R, Nichols L, Ann Teale E, et al. Development and validation of an electronic frailty index using routine primary care electronic health record data. Age Ageing. 2016;45(3):353–60. 10.1093/ageing/afw039 PMC4846793 26944937

[30] Mitnitski AB, Mogilner AJ, Rockwood K. Accumulation of deficits as a proxy measure of aging. Sci World J. 2001;1:323–36. 10.1100/tsw.2001.58 PMC6084020 12806071

[31] NHS England. NHS England Standard General Medical Services Contract 2017/18. 2018. Available from: https://www.england.nhs.uk/publication/nhs-england-standard-general-medical-services-contract-2017-18/. [cited 2025 22 July].

[32] Jünger S, Payne SA, Brine J, Radbruch L, Brearley SG. Guidance on Conducting and REporting DElphi Studies (CREDES) in palliative care: Recommendations based on a methodological systematic review. Palliat Med. 2017;31(8):684–706. 10.1177/0269216317690685 28190381

[33] Birko S, Dove ES, Özdemir V. Evaluation of Nine Consensus Indices in Delphi Foresight Research and Their Dependency on Delphi Survey Characteristics: A Simulation Study and Debate on Delphi Design and Interpretation. PLoS ONE. 2015;10(8):e0135162. 10.1371/journal.pone.0135162 PMC4535950 26270647

[34] Chalmers J, Armour M. The Delphi Technique. In: Liamputtong P, editor. Handbook of Research Methods in Health Social Sciences. Singapore: Springer Singapore; 2019. pp. 715–35.

[35] Beiderbeck D, Frevel N, von der Gracht HA, Schmidt SL, Schweitzer VM. Preparing, conducting, and analyzing Delphi surveys: Cross-disciplinary practices, new directions, and advancements. MethodsX. 2021;8:101401. 10.1016/j.mex.2021.101401 PMC8374446 34430297

[36] Garritty C, Gartlehner G, Nussbaumer-Streit B, King VJ, Hamel C, Kamel C, et al. Cochrane Rapid Reviews Methods Group offers evidence-informed guidance to conduct rapid reviews. J Clin Epidemiol. 2021;130:13–22. 10.1016/j.jclinepi.2020.10.007 PMC7557165 33068715

[37] Davis FD, Bagozzi RP, Warshaw PR. User Acceptance of Computer Technology: A Comparison of Two Theoretical Models. Manage Sci. 1989;35(8):982–1003.

[38] Ahuja S, Gronholm PC, Shidhaye R, Jordans M, Thornicroft G. Development of mental health indicators at the district level in Madhya Pradesh, India: mixed methods study. BMC Health Serv Res. 2018;18(1):867. 10.1186/s12913-018-3695-4 PMC6245827 30453960

[39] Huang J-Y, Hsu H-C, Hsiao Y-L, Chen F-Y, Lo S-Y, Chou T-Y et al. Developing Indicators of Age-Friendliness in Taiwanese Communities through a Modified Delphi Method. International Journal of Environmental Research and Public Health [Internet]. 2022; 19(21). Available from: https://mdpi-res.com/d_attachment/ijerph/ijerph-19-14430/article_deploy/ijerph-19-14430-v2.pdf?version=1667783522 10.3390/ijerph192114430 PMC9658640 36361310

[40] Jiang F, Liu T, Zhou H, Rakofsky JJ, Liu H, Liu Y, et al. Developing medical record-based, healthcare quality indicators for psychiatric hospitals in China: a modified Delphi-Analytic Hierarchy Process study. Int J Qual Health Care. 2019;31(10):733–40. 10.1093/intqhc/mzz005 30753601

[41] Tang X, Chen X, Pang Y, Zhou L. The development of quality indicators for home care in China. Int J Qual Health Care. 2018;30(3):208–18. 10.1093/intqhc/mzx202 29385451

[42] Campbell M, Stewart T, Brunkert T, Campbell-Enns H, Gruneir A, Halas G, et al. Prioritizing supports and services to help older adults age in place: A Delphi study comparing the perspectives of family/friend care partners and healthcare stakeholders. PLoS ONE. 2021;16(11):e0259387. 10.1371/journal.pone.0259387 PMC8577765 34752475

[43] Abe T, Carver A, Sugiyama T. Associations of neighborhood built and social environments with frailty among mid-to‐older aged Australian adults. Geriatr Gerontol Int. 2021;21(10):893–9. 10.1111/ggi.14253 34355479

[44] Fritz H, Cutchin MP, Gharib J, Haryadi N, Patel M, Patel N. Neighborhood Characteristics and Frailty: A Scoping Review. Gerontologist. 2020;60(4):e270–85. 10.1093/geront/gnz072 PMC7768736 31276582

[45] Goto T, Kishimoto T, Fujiwara S, Shirayama Y, Ichikawa T. Social frailty as a predictor of all-cause mortality and functional disability: a systematic review and meta-analysis. Sci Rep. 2024;14(1):3410. 10.1038/s41598-024-53984-3 PMC10858956 38341512

[46] Mori Y, Tsuji T, Watanabe R, Hanazato M, Miyazawa T, Kondo K. Built environments and frailty in older adults: A three-year longitudinal JAGES study. Arch Gerontol Geriatr. 2022;103:104773. 10.1016/j.archger.2022.104773 35849975

[47] Qiu Y, Li G, Zheng L, Liu W, Li X, Wang X, et al. Relationship Between Cognitive Frailty and Mortality in Older Adults: A Systematic Review and Meta-Analysis. J Am Med Dir Assoc. 2023;24(11):1637–e448. 10.1016/j.jamda.2023.08.001 37660724

[48] Uemura K, Kamitani T, Yamada M. Frailty and Environmental Attributes in Older Adults: Insight from an Ecological Model. Phys Therapy Res. 2023;26(3):71–7. 10.1298/ptr.R0027 PMC10730123 38125292

[49] Ye L, van Grieken A, Alhambra-Borrás T, Zhou S, Clough G, Markaki A, et al. Interplay of Physical, Psychological, and Social Frailty among Community-Dwelling Older Adults in Five European Countries: A Longitudinal Study. J Urb Health. 2024;101(4):730–9. 10.1007/s11524-024-00831-5 PMC11329455 38913271

[50] Zhang X-M, Cao S, Gao M, Xiao S, Xie X, Wu X. The Prevalence of Social Frailty Among Older Adults: A Systematic Review and Meta-Analysis. J Am Med Dir Assoc. 2023;24(1):29–e379. 10.1016/j.jamda.2022.10.007 36402197

[51] Zhao J, Liu YWJ, Tyrovolas S, Mutz J. Exploring the concept of psychological frailty in older adults: a systematic scoping review. J Clin Epidemiol. 2023;159:300–8. 10.1016/j.jclinepi.2023.05.005 37156339

[52] Gell NM, Rosenberg DE, Demiris G, LaCroix AZ, Patel KV. Patterns of Technology Use Among Older Adults With and Without Disabilities. Gerontologist. 2015;55(3):412–21. 10.1093/geront/gnt166 PMC4542705 24379019

[53] Keränen NS, Kangas M, Immonen M, Similä H, Enwald H, Korpelainen R, Jämsä T. Use of Information and Communication Technologies Among Older People With and Without Frailty: A Population-Based Survey. J Med Internet Res. 2017;19(2):e29. 10.2196/jmir.5507. PMC5331186 28196791

[54] Lee DR, Lo JC, Ramalingam N, Gordon NP. Understanding the Uptake of Digital Technologies for Health-Related Purposes in Frail Older Adults. J Am Geriatr Soc. 2021;69(1):269–72. 10.1111/jgs.16841 33011975

[55] Shiratsuchi D, Makizako H, Akaida S, Tateishi M, Hirano H, Iijima K, et al. Associations between information and communication technology use and frailty in community-dwelling old-old adults: results from the ILSA-J. Eur Geriatr Med. 2024;15:621–7. 10.1007/s41999-024-00979-y PMC11329410 38722515

[56] Age UK. Offline and Overlooked: Digital exclusion and its impact on older people. 2024. Available from: https://www.ageuk.org.uk/siteassets/documents/reports-and-publications/reports-and-briefings/offline-and-overlooked-report.pdf. Cited 2025 26 July.

[57] Cruz AM, Monsalve LM, Ladurner A-ML, Jaime LFJ, Wang DW, Quiroga DAQ. Information and Communication Technologies for Managing Frailty: A Systematic Literature Review. Aging Disease. 2021;12(3):914–33. 10.14336/AD.2020.1114 PMC8139198 34094651

[58] Linn N, Goetzinger C, Regnaux J-P, Schmitz S, Dessenne C, Fagherazzi G et al. Digital Health Interventions among People Living with Frailty: A Scoping Review. J Am Med Dir Assoc. 2021;22(9):1802-12.e21. 10.1016/j.jamda.2021.04.012 34000266

[59] Pontin FL, Jenneson VL, Morris MA, Clarke GP, Lomax NM. Objectively measuring the association between the built environment and physical activity: a systematic review and reporting framework. Int J Behav Nutr Phys Act. 2022;19(1):1–119. 10.1186/s12966-022-01352-7 PMC9476279 36104757

[60] Zhou J-J, Kang R, Bai X. A Meta-Analysis on the Influence of Age-Friendly Environments on Older Adults’ Physical and Mental Well-Being. Int J Environ Res Public Health. 2022;19(21):13813. 10.3390/ijerph192113813 PMC9657613 36360692

[61] Honvo G, Sabico S, Veronese N, Bruyère O, Rizzoli R, Amuthavalli Thiyagarajan J, et al. Measures of attributes of locomotor capacity in older people: a systematic literature review following the COSMIN methodology. Age Ageing. 2023;52(Supplement_4):iv44–66. 10.1093/ageing/afad139 PMC10615073 37902521

[62] Julião PL, Fernandes ÓB, Alves JP, Amuthavalli Thiyagarajan J, Mikton C, Diaz T, et al. A systematic review of reviews on the psychometric properties of measures of older persons’ ability to build and maintain social relationships. Age Ageing. 2023;52(Supplement_4):iv133–7. 10.1093/ageing/afad106 PMC10615056 37902525

[63] Knoop V, Mathot E, Louter F, Beckwee D, Mikton C, Diaz T, et al. Measurement properties of instruments to measure the fatigue domain of vitality capacity in community-dwelling older people: an umbrella review of systematic reviews and meta-analysis. Age Ageing. 2023;52(Supplement_4):iv26–43. 10.1093/ageing/afad140 PMC10615047 37902527

[64] Kuspinar A, Mehdipour A, Beauchamp MK, Hao Q, Cino E, Mikton C, et al. Assessing the measurement properties of life-space mobility measures in community-dwelling older adults: a systematic review. Age Ageing. 2023;52(Supplement_4):iv86–99. 10.1093/ageing/afad119 PMC10615067 37902523

[65] Mansor N, Awang H, Amuthavalli Thiyagarajan J, Mikton C, Diaz T. Measures of ability to learn, grow and make decisions among older persons: a systematic review of measurement properties. Age Ageing. 2023;52(Supplement_4):iv118–32. 10.1093/ageing/afad101 PMC10615040 37902520

[66] Oster C, Hines S, Rissel C, Asante D, Khadka J, Seeher KM, et al. A systematic review of the measurement properties of aspects of psychological capacity in older adults. Age Ageing. 2023;52(Supplement_4):iv67–81. 10.1093/ageing/afad100 PMC10615039 37902524

[67] Williams T, Geffen L, Kalula S, Stein DJ, Amuthavalli Thiyagarajan J, Mikton C, et al. A systematic review of measures of ability to meet basic needs in older persons. Age Ageing. 2023;52(Supplement_4):iv112–7. 10.1093/ageing/afad121 PMC10615038 37902519

[68] Saelens BE, Sallis JF, Black JB, Chen D. Neighborhood-Based Differences in Physical Activity: An Environment Scale Evaluation. Am J Public Health. 2003;93(9):1552–8. 10.2105/ajph.93.9.1552 PMC1448009 12948979

[69] Chen K, Lou VWQ. Measuring Senior Technology Acceptance: Development of a Brief, 14-Item Scale. Innov Aging. 2020;4(3):igaa016. 10.1093/geroni/igaa016 PMC7320876 32617418

[70] Maharani A, Sinclair DR, Chandola T, Bower P, Clegg A, Hanratty B, et al. Household wealth, neighbourhood deprivation and frailty amongst middle-aged and older adults in England: a longitudinal analysis over 15 years (2002–2017). Age Ageing. 2023;52(3):afad034. 10.1093/ageing/afad034 PMC10061942 36995138

[71] Sinclair DR, Maharani A, Chandola T, Bower P, Hanratty B, Nazroo J, et al. Frailty among Older Adults and Its Distribution in England. J Frailty Aging. 2022;11(2):163–8. 10.14283/jfa.2021.55 35441193

[72] Straughan P, Tan YW, Tiew Z, Zheng Z, Ngu R, Hiah WT. Ageing in Place—The Key to Receiving a Superaged Society. Populations [Internet]. 2025; 1(2). Available from: https://mdpi-res.com/d_attachment/populations/populations-01-00007/article_deploy/populations-01-00007.pdf?version=1744030917

[73] The King’s Fund. Key facts and figures about adult social care. 2025 [updated 19 June 2025; cited 2025 22 July]. Available from: https://www.kingsfund.org.uk/insight-and-analysis/data-and-charts/key-facts-figures-adult-social-care#how-much….

[74] Homecare Association. LaingBuisson adult social care market report. 2024 [cited 2025 22 July]. Available from: https://www.homecareassociation.org.uk/resource/laingbuisson-adult-social-care-market-report.html

[75] Age UK. Paying for homecare. 2025 [updated 15 April 2025; cited 2025 22 July]. Available from: https://www.ageuk.org.uk/information-advice/care/paying-for-care/paying-for-homecare/

[76] Nikolova S, Heaven A, Hulme C, West R, Pendleton N, Humphrey S, et al. Social care costs for community-dwelling older people living with frailty. Health Soc Care Commun. 2022;30(3):e804–11. 10.1111/hsc.13450 34080751

[77] Walsh B, Fogg C, England T, Brailsford S, Roderick P, Harris S et al. Impact of frailty in older people on health care demand: simulation modelling of population dynamics to inform service planning. Health Soc Care Deliv Res. 2024;12(44). 10.3310/LKJF3976. 39487824

[78] Graham L, Brundle C, Harrison N, Andre D, Clegg A, Forster A, et al. What are the priorities for research of older people living in their own home, including those living with frailty? A systematic review and content analysis of studies reporting older people’s priorities and unmet needs. Age Ageing. 2024;53(1):afad232. 10.1093/ageing/afad232 PMC10798941 38243402

[79] Kwan I, Rutter D, Anderson B, Stansfield C. Personal care and practical support at home: a systematic review of older people’s views and experiences. Working Older People. 2019;23(2):87–106.

[80] O’Rourke G, Beresford B. Research priorities for homecare for older people: A UK multi-stakeholder consultation. Health Soc Care Commun. 2022;30(6):e5647–60. 10.1111/hsc.13991 PMC10087309 36134898

[81] Best K, Shuweihdi F, Alvarez JCB, Relton S, Avgerinou C, Nimmons D, et al. Development and external validation of the electronic frailty index 2 using routine primary care electronic health record data. Age Ageing. 2025;54(4):afaf077. 10.1093/ageing/afaf077 PMC11957239 40163740

[82] Romero-Ortuno R, Keevil VL. Advancing frailty identification in UK primary care with eFI2: are we there yet? Age Ageing. 2025;54(5):afaf111. 10.1093/ageing/afaf111 40319482

[83] Department of Health and Social Care. Data saves lives: reshaping health and social care with data: Department of Health & Social Care.; 2022 [updated 15 August 2022; cited 2025 22 July]. Available from: https://www.gov.uk/government/publications/data-saves-lives-reshaping-health-and-social-care-with-data/data-saves-lives-reshaping-health-and-social-care-with-data.

[84] NHS England. Improving support for people with frailty through Integrated Neighbourhood Teams.2023 [updated 20 September 2023; cited 2025 22 July]. Available from: https://www.england.nhs.uk/long-read/improving-support-for-people-with-frailty-through-integrated-neighbourhood-teams/

[85] NHS England. Warwickshire frailty service keeps half of patients at home after falls. 2025 [cited 2025 22 July]. Available from: https://www.england.nhs.uk/integratedcare/resources/case-studies/warwickshire-frailty-service-keeps-half-of-patients-at-home-after-falls/

[86] NHS England. Working with communities to mobilise change through neighbourhood multidisciplinary teams (frailty focused). 2025 [updated 5 March 2025; cited 2025 22 July]. Available from: https://www.england.nhs.uk/long-read/working-with-communities-to-mobilise-change-through-neighbourhood-multidisciplinary-teams-frailty-focused/

[87] Bian C, Ye B, Hoonakker A, Mihailidis A. Attitudes and perspectives of older adults on technologies for assessing frailty in home settings: a focus group study. BMC Geriatr. 2021;21(1):298. 10.1186/s12877-021-02252-4 PMC8105977 33964887

[88] Ollevier A, Aguiar G, Palomino M, Simpelaere IS. How can technology support ageing in place in healthy older adults? A systematic review. Public Health Rev. 2020;41(1):26. 10.1186/s40985-020-00143-4 PMC7684947 33292707

[89] Wang J, Du Y, Coleman DC, Peck M, Myneni S, Kang H, et al. Mobile and Connected Health Technology Needs for Older Adults Aging in Place: Cross-Sectional Survey Study. JMIR Aging. 2019;2:e13864. 10.2196/13864 PMC6715003 31518283

[90] Urtamo A, Jyväkorpi SK, Strandberg TE. Definitions of successful ageing: A brief review of a multidimensional concept. Acta bio-medica de l’Ateneo Parmense. 2019;90(2):359–63. 10.23750/abm.v90i2.8376 PMC6776218 31125022

[91] Winkler J, Moser R. Biases in future-oriented Delphi studies: A cognitive perspective. Technol Forecast Soc Chang. 2016;105:63–76.

