# Supplementary material for: Proxy indicators to support independent ageing-in-place in older people with frailty: a Delphi-style expert consultation survey
Source: BMC Geriatr. 2026 Jul 28;26:1335. doi: 10.1186/s12877-026-07968-9 (PMC13693169; doi:10.1186/s12877-026-07968-9)
Supplement: Supplementary file 1 [file 12877_2026_7968_MOESM4_ESM.pdf]

Ageing-in-Place Indicators for Older People with Frailty

An Expert Consultation Survey

What was the aim?

To identify indicators that would help service providers and carers understand how older people with frailty are ageing in their homes and communities (that is, how they are *ageing in place*). This would help prevent hospitalisations and transitions to care homes.

Who were the experts?

11 Academics and Researchers

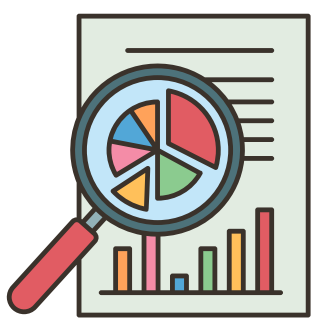

5 Healthcare Practitioners

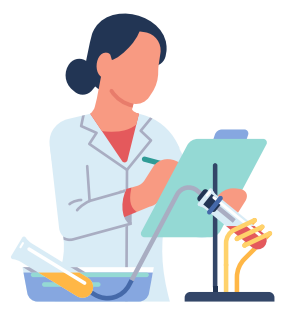

2 Social Care and Policy Professionals

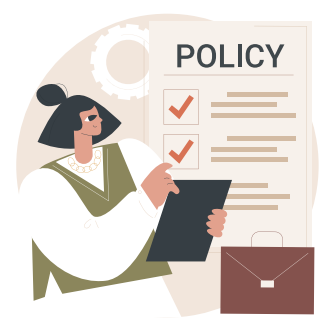

What did the experts do?

Experts completed two surveys. They rated each indicator from 1 to 5 across two criteria:

Importance: how important is this indicator to understand how older people with frailty are ageing in place?

Feasibility: how feasibly can this indicator be routinely measured in health and social care practice?

We calculated the average scores for each indicator in the first survey. In the second survey, experts could change their scores to align with this mean score of each indicator.

What were the indicators?

We extracted 89 indicators from current research across five categories:

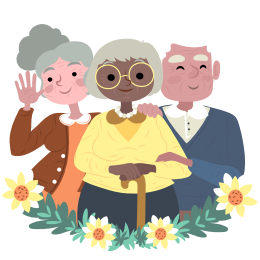

Personal characteristics of older people

41 indicators

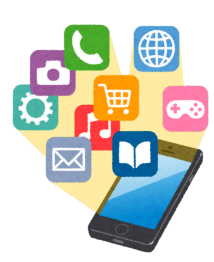

Technology

14 indicators

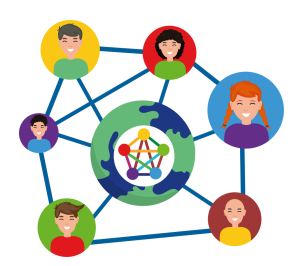

Social networks

8 indicators

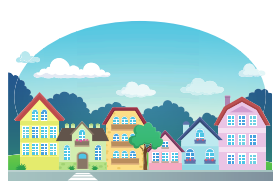

Place

18 indicators

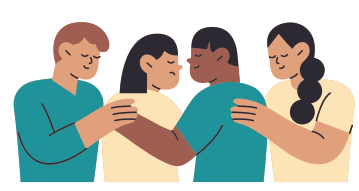

Formal and informal support

8 indicators

What were the key results?

TOP-RANKED INDICATORS

Only 4 indicators were extremely / very important for ageing-in-place ***and*** extremely / very feasible to routinely measure:

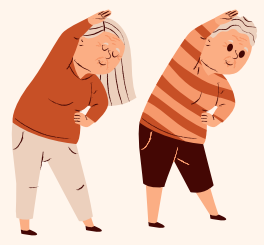

Physical performance and mobility

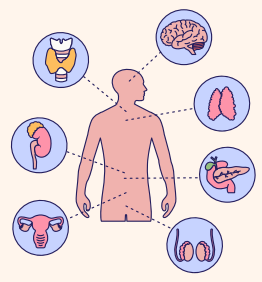

Having multiple chronic conditions

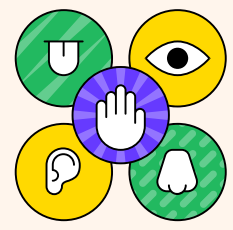

Sensory function

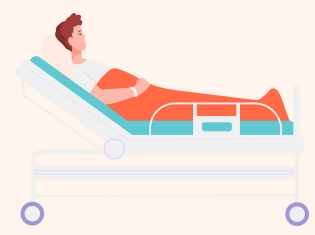

Pressure ulcers

IMPORTANT BUT NOT FEASIBLE INDICATORS

27 indicators were extremely / very important for ageing-in-place, but not feasible to routinely measure.

20 Personal characteristics

4 Social networks

3 Place & Support

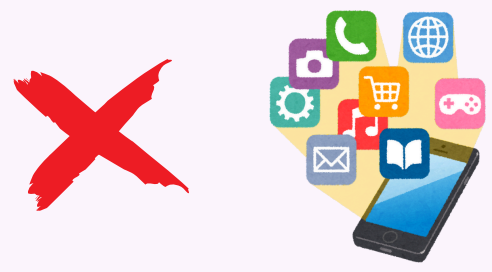

No indicators related to **technology** were extremely / very important or feasible to routinely measure.

HIGEST-RATED INDICATORS BY EXPERTS

8 personal characteristics were extremely / very important for ageing-in-place by ***all*** experts:

- **Self-care ability**
- **Sensory function**
- **Falls-related indicators**
- **Physical activity participation**
- **Social vulnerability**
- **Physical performance and mobility**
- **Independence and confidence in activities of daily living**
- **Memory and cognition**

Extremely **important for ageing-in-place by most experts**

Only "Physical performance and mobility" was extremely / very feasible to routinely measure by ***all*** experts.

What are the future directions?

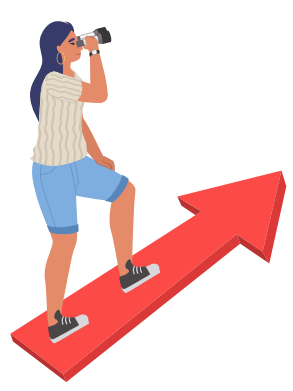

- Explore feasibility of important but hard-to-measure indicators.
- Develop strategies to integrate ageing-in-place indicators into routine assessments.
- Use these indicators to deliver tailored care to older people with frailty.

What do we want to learn from you?

What are your thoughts on the survey results?

Do you generally agree with the experts' opinions?

What are your thoughts / experiences on routinely measuring these indicators? (from a service user perspective)

What indicators do YOU think are most crucial for ageing in place?

How can we take this research forward?

What are the details of the research?

This research is part of a PhD project, based in the School of Health Sciences in the University of Manchester.

PhD researcher: Schenelle Dlima (schenelle.dlima@postgrad.manchester.ac.uk)

Main supervisor: Prof Emma Vardy

The project has been reviewed by The University of Manchester Proportionate Research Ethics Committee (reference: 2024-18943-36066).
