# Supplementary material for: Proxy indicators to support independent ageing-in-place in older people with frailty: a Delphi-style expert consultation survey
Source: BMC Geriatr. 2026 Jul 28;26:1335. doi: 10.1186/s12877-026-07968-9 (PMC13693169; doi:10.1186/s12877-026-07968-9)
Supplement: Supplementary file 2 [file 12877_2026_7968_MOESM1_ESM.docx]

Supplement 1: Search strategy for rapid review to identify potential ageing-in-place indicators

# S1.1. Eligibility criteria

The broad inclusion criteria were as follows:

- If studies included older adults aged ≥50 years living in their homes or in the community.
- Observational studies where data were collected from the study participants using self-reports or objective methods.
- Reviews that described conceptualisations and frameworks for ageing-in-place in older people.
- Systematic reviews that evaluated interventions for community-dwelling older adults with frailty, and risk factors for institutionalisation in older people.
- Trials and trial protocols related to frailty in community-dwelling older people and ageing-in-place

The broad exclusion criteria were as follows:

- Conference abstracts, opinion pieces, review protocols.
- Older people in institutional settings, such as nursing, care, and residential homes.
- Publications focusing on specific conditions other than frailty.

# S1.2. Search terms used for each database

All searches were conducted in December 2023.

## Medline (Ovid)

| 1 | (indicator* or measure* or proxy or candidate).mp. |
| --- | --- |
| 2 | frail*.mp. |
| 3 | *Frailty/ or *Frail Elderly/ |
| 4 | (aging in place or age in place or ageing in place or age at home or ageing at home or aging at home).ab,ti. |
| 5 | *independent living/ |
| 6 | 2 or 3 |
| 7 | 1 or 6 |
| 8 | 4 or 5 |
| 9 | 7 and 8 |

------------------------------------------------------------------------------------------------------------

## Embase

| 1 | (indicator* or measure* or proxy or candidate).mp. |
| --- | --- |
| 2 | frail*.mp. |
| 3 | *frail elderly/ |
| 4 | *frailty/ |
| 5 | (aging in place or age in place or ageing in place or age at home or ageing at home or aging at home).ab,ti. |
| 6 | *independent living/ |
| 7 | 2 or 3 or 4 |
| 8 | 1 or 7 |
| 9 | 5 or 6 |
| 10 | 8 and 9 |

------------------------------------------------------------------------------------------------------------

## PsycINFO

| 1 | exp Aging in Place/ |
| --- | --- |
| 2 | (aging in place or age in place or ageing in place or age at home or ageing at home or aging at home).ab,ti. |
| 3 | (indicator* or measure* or proxy or candidate).mp. |
| 4 | frail*.mp. |
| 5 | 1 or 2 |
| 6 | 3 or 4 |
| 7 | 5 and 6 |

------------------------------------------------------------------------------------------------------------

## Cochrane Trials

In title, abstract, and keywords, with search word variation allowed:

**Ageing-in-place**

**AND**

**Frail***

------------------------------------------------------------------------------------------------------------

# S1.3. Flowchart for publication selection process

A total of 340 full records were screened to identify and extract potential ageing-in-place indicators.

**Identification of studies via databases**

Reasons for exclusion (n = 507):

- Conference abstracts, opinion pieces, commentaries (n = 222)
- Protocol (n = 5)
- Frailty not measured using FI (n = 85)
- FI not constructed from routine data (n = 55)
- FI not presented as a proportion (n = 51)
- Frailty assessment tool not specified (n = 25)
- FI was not the main tool investigated or used to measure frailty (n = 13)
- Not in English (n = 8)
- Full text not found (n = 43)

Records identified (n = 3,725):

Medline (Ovid) = 2,318

Embase = 1,144

Medline = 1,295

PsycInfo = 202

Cochrane Trials = 61

Records after de-duplication

(n = 2,685)

Records screened to extract ageing-in-place indicators

(n = 340)

Records screened for titles, abstracts, and keywords

(n = 2,685)

**Screening**

**Identification**
