# Supplementary material for: Proxy indicators to support independent ageing-in-place in older people with frailty: a Delphi-style expert consultation survey
Source: BMC Geriatr. 2026 Jul 28;26:1335. doi: 10.1186/s12877-026-07968-9 (PMC13693169; doi:10.1186/s12877-026-07968-9)
Supplement: Supplementary file 3 [file 12877_2026_7968_MOESM3_ESM.docx]

Supplement 3: Survey questionnaire distributed to experts

____________________________________________________________________________

# Section 1: Introduction before survey start

You are invited to take part in this two-stage online survey about identifying potential indicators of ageing-in-place in community-dwelling older people with frailty in England. This survey is part of a PhD research project.

This survey will take approximately 80 minutes to complete. You can return to the survey at a later time, and your responses will be saved.

If you have any questions, please email ________ [email address of first author was shared].

Before proceeding, we kindly ask you to read the following and confirm your consent to participate:

- I confirm that I have read the participant information sheet (Version 6, Date 09/07/2024) for the above study and have had the opportunity to consider the information and ask questions and had these answered satisfactorily. You can download the participant information sheet here [link to downloadable word document was provided].
- I understand that my participation in the study is voluntary and that I am free to withdraw at any time without giving a reason and without detriment to myself.
- I agree that any data collected may be included in anonymous form in publications/conference presentations.
- I understand that a fully anonymised dataset may be deposited in an open data repository at the end of the project.
- I understand that my email address will be retained by the research team to circulate summary reports and outputs.
- I understand that data collected during the study may be looked at by individuals from The University of Manchester or regulatory authorities, where it is relevant to my taking part in this research. I give permission for these individuals to have access to my data.

I consent to take part in this survey.

- Yes
- No

I am able to take part in both rounds of the survey.

- Yes
- No

# Section 2: Participant information

Please complete the following questions on your demographic and professional background.

**1. Age (in years):**

- <25
- 25–34
- 35–44
- 45–54
- 55–64
- ≥65
- Prefer not to say

**2. In which geographical region is your place of work or institution?** You can refer to this link for clarification: https://www.ons.gov.uk/methodology/geography/ukgeographies/eurostat

- North East England
- North West England
- Yorkshire and The Humber
- East Midlands
- West Midlands
- East England
- London
- South East England
- South West England
- Scotland
- Northern Ireland
- Prefer not to say

**3. Which of the following best describes your gender?**

- Man
- Woman
- Non-binary
- My gender is not listed
- Prefer not to say

**4. What is your ethnic group?**

- Asian / Asian British
- Black / African / Caribbean / Black British
- Mixed / Multiple ethnic groups
- White
- Other ethnic group (feel free to specify below)

[Free text response]

- Prefer not to say

**5. Current job title:**

[Free text response]

**6. For how long in your career have you focused on care and/or research related to older people and ageing?**

- <5 years
- 5–15 years
- 16–25 years
- >25 years

**7. How do you describe your area of expertise? (for example, hospital care, community care, etc.)**

[Free text response]

8**. Highest qualification:**

- Bachelor's degree
- Master's degree
- Medical degree (BMBS, BM, MBChB)
- Doctorate (PhD) degree
- Other professional degree (please specify below)

[Free text response]

- Prefer not to say

# Section 3: Personal characteristics of older people

This section includes 41 indicators related to sociodemographic, clinical, behavioural, and interpersonal characteristics of older people with or at risk of frailty.

We have provided a brief description of each indicator. We ask you to rate each indicator according to two criteria:

Importance: how important is this indicator in understanding how older people with frailty or at risk of frailty are ageing in place?

1 – "not at all important"

2 – "somewhat important"

3 – "quite important"

4 – "very important"

5 – "extremely important"

Feasibility: how feasibly can this indicator be routinely measured in health and social care practice?

1 – "not at all feasible"

2 – "somewhat feasible"

3 – "quite feasible"

4 – "very feasible"

5 – "extremely feasible"

You are also provided with free text boxes below each indicator to add your comments for that indicator. Please refrain from completing the free text boxes with potentially identifiable information such as names, places, etc.

Please note that rating scale responses for all indicators are required, but adding comments is optional. You can return to the survey at any time using the same link, and your responses will be saved.

**1. Physical activity participation**

Doing some form of aerobic activity (like brisk walking, climbing stairs, etc.) or strength-improving activity (like carrying or moving loads such as groceries, weight training, chair-based exercises, etc.).

|  | 1 (not at all) | 2 (somewhat) | 3 (quite) | 4 (very) | 5 (extremely) |
| --- | --- | --- | --- | --- | --- |
| Important for ageing-in-place |  |  |  |  |  |
| Feasible to routinely measure |  |  |  |  |  |

Comments about indicator *physical activity participation***:**

[Free text response]

**2. Self-perceived health**

How a person rates their physical, social, emotional, and spiritual health.

|  | 1 (not at all) | 2 (somewhat) | 3 (quite) | 4 (very) | 5 (extremely) |
| --- | --- | --- | --- | --- | --- |
| Important for ageing-in-place |  |  |  |  |  |
| Feasible to routinely measure |  |  |  |  |  |

Comments about indicator *self-perceived health***:**

[Free text response]

**3. Fatigue and tiredness**

The energy, enthusiasm, and endurance that a person has to do daily tasks and recreational activities.

|  | 1 (not at all) | 2 (somewhat) | 3 (quite) | 4 (very) | 5 (extremely) |
| --- | --- | --- | --- | --- | --- |
| Important for ageing-in-place |  |  |  |  |  |
| Feasible to routinely measure |  |  |  |  |  |

Comments about indicator *fatigue and tiredness***:**

[Free text response]

**4. Physical performance and mobility**

Indicators may include:

- Muscle power and strength
- If they can move easily from one position to another (such as standing up from a chair)
- Walking or gait speed
- Balance, i.e. being able to stand unsupported with their feet in a certain position

|  | 1 (not at all) | 2 (somewhat) | 3 (quite) | 4 (very) | 5 (extremely) |
| --- | --- | --- | --- | --- | --- |
| Important for ageing-in-place |  |  |  |  |  |
| Feasible to routinely measure |  |  |  |  |  |

Comments about indicator *physical performance and mobility*:

[Free text response]

**5. Independence and confidence in undertaking activities of daily living**

Whether the person can carry out these tasks (objective), or how confident or secure the person feels in doing these tasks (subjective):

- Eating food
- Bathing
- Grooming
- Dressing
- Using the toilet
- Bowel control
- Bladder control
- Transferring (bed to chair and back)
- Mobility on level surfaces
- Using the stairs

|  | 1 (not at all) | 2 (somewhat) | 3 (quite) | 4 (very) | 5 (extremely) |
| --- | --- | --- | --- | --- | --- |
| Important for ageing-in-place |  |  |  |  |  |
| Feasible to routinely measure |  |  |  |  |  |

Comments about indicator *independence and confidence in undertaking activities of daily living*:

[Free text response]

**6. Independence and confidence in undertaking instrumental activities of daily living**

Whether the person can carry out these tasks (objective), or how confident or secure the person feels in doing these tasks (subjective):

- Cooking
- Cleaning
- Transportation
- Laundry
- Managing finances

|  | 1 (not at all) | 2 (somewhat) | 3 (quite) | 4 (very) | 5 (extremely) |
| --- | --- | --- | --- | --- | --- |
| Important for ageing-in-place |  |  |  |  |  |
| Feasible to routinely measure |  |  |  |  |  |

Comments about indicator *independence and confidence in undertaking instrumental activities of daily living*:

[Free text response]

**7. Quality of life**

How someone sees their life in the context of their culture and values, and how it fits with their goals, expectations, and worries. Indicators could include:

- How happy someone is with their health, surroundings, support from others, and getting information they need
- Pain
- Sleep
- Energy levels
- Negative feelings and mood

|  | 1 (not at all) | 2 (somewhat) | 3 (quite) | 4 (very) | 5 (extremely) |
| --- | --- | --- | --- | --- | --- |
| Important for ageing-in-place |  |  |  |  |  |
| Feasible to routinely measure |  |  |  |  |  |

Comments about indicator *quality of life*:

[Free text response]

**8. Loneliness**

Feeling unhappy with the frequency and quality of social relationships.

|  | 1 (not at all) | 2 (somewhat) | 3 (quite) | 4 (very) | 5 (extremely) |
| --- | --- | --- | --- | --- | --- |
| Important for ageing-in-place |  |  |  |  |  |
| Feasible to routinely measure |  |  |  |  |  |

Comments about indicator *loneliness*:

[Free text response]

**9. Body composition**

Using dual X-ray absorptiometry (DXA) or bioelectrical impedance analysis (BIA), this can include measuring:

- Skeletal muscle mass
- Body fat mass
- Total body water
- Basal metabolic rate
- Bone density

|  | 1 (not at all) | 2 (somewhat) | 3 (quite) | 4 (very) | 5 (extremely) |
| --- | --- | --- | --- | --- | --- |
| Important for ageing-in-place |  |  |  |  |  |
| Feasible to routinely measure |  |  |  |  |  |

Comments about indicator *body composition*:

[Free text response]

**10. Anthropometry**

This includes measuring:

- Bodyweight
- Body mass index (BMI)
- Waist, calf, and mid-upper arm circumference
- Grip strength
- Skinfold thickness

|  | 1 (not at all) | 2 (somewhat) | 3 (quite) | 4 (very) | 5 (extremely) |
| --- | --- | --- | --- | --- | --- |
| Important for ageing-in-place |  |  |  |  |  |
| Feasible to routinely measure |  |  |  |  |  |

Comments about indicator *anthropometry*:

[Free text response]

**11. Pain**

Unpleasant physical sensations, and how much they bother the person and get in the way of doing things.

|  | 1 (not at all) | 2 (somewhat) | 3 (quite) | 4 (very) | 5 (extremely) |
| --- | --- | --- | --- | --- | --- |
| Important for ageing-in-place |  |  |  |  |  |
| Feasible to routinely measure |  |  |  |  |  |

Comments about indicator *pain*:

[Free text response]

**12. Social vulnerability**

This could include:

- Being widowed or separated
- Housing problems
- Living alone with no help available
- Few or infrequent social contacts

|  | 1 (not at all) | 2 (somewhat) | 3 (quite) | 4 (very) | 5 (extremely) |
| --- | --- | --- | --- | --- | --- |
| Important for ageing-in-place |  |  |  |  |  |
| Feasible to routinely measure |  |  |  |  |  |

Comments about indicator *social vulnerability*:

[Free text response]

**13. Oral health status**

How well a person takes care of their oral hygiene every day. Indicators could include:

- Swallowing and chewing ability
- Gum health
- Number of natural and functioning teeth
- Tongue function
- Signs such as dry mouth and bad breath

|  | 1 (not at all) | 2 (somewhat) | 3 (quite) | 4 (very) | 5 (extremely) |
| --- | --- | --- | --- | --- | --- |
| Important for ageing-in-place |  |  |  |  |  |
| Feasible to routinely measure |  |  |  |  |  |

Comments about indicator *oral health status*:

[Free text response]

**14. Falls-related indicators**

These can include:

- How often the person falls.
- Being scared of falling, which might stop them from doing things.
- How confident the person feels in doing tasks without falling.

|  | 1 (not at all) | 2 (somewhat) | 3 (quite) | 4 (very) | 5 (extremely) |
| --- | --- | --- | --- | --- | --- |
| Important for ageing-in-place |  |  |  |  |  |
| Feasible to routinely measure |  |  |  |  |  |

Comments about indicator *falls-related indicators*:

[Free text response]

**15. Financial security**

What a person can afford or cannot afford, which might affect their quality of life and access to services.

|  | 1 (not at all) | 2 (somewhat) | 3 (quite) | 4 (very) | 5 (extremely) |
| --- | --- | --- | --- | --- | --- |
| Important for ageing-in-place |  |  |  |  |  |
| Feasible to routinely measure |  |  |  |  |  |

Comments about indicator *financial security*:

[Free text response]

**16. Nutritional status**

Whether the person is at risk of malnutrition based on:

- Weight loss
- Appetite loss and reduced food intake
- Dehydration levels
- Body mass index
- Calf, waist, or mid-upper arm circumference
- Functional loss due to muscle wasting

|  | 1 (not at all) | 2 (somewhat) | 3 (quite) | 4 (very) | 5 (extremely) |
| --- | --- | --- | --- | --- | --- |
| Important for ageing-in-place |  |  |  |  |  |
| Feasible to routinely measure |  |  |  |  |  |

Comments about indicator *nutritional status*:

[Free text response]

**17. Dietary patterns**

These can include:

- Adequate intake of foods in specific food groups
- Portion sizes
- Number of meals a day
- Nutrient intake (proteins, carbohydrates, fats, vitamins, minerals, etc.)
- Food choices
- Food quality
- Access to culturally appropriate food
- Access to balanced diet suitable to their culture

|  | 1 (not at all) | 2 (somewhat) | 3 (quite) | 4 (very) | 5 (extremely) |
| --- | --- | --- | --- | --- | --- |
| Important for ageing-in-place |  |  |  |  |  |
| Feasible to routinely measure |  |  |  |  |  |

Comments about indicator *dietary patterns*:

[Free text response]

**18. Self-care ability**

How well a person can start and keep up with activities that help them stay healthy and happy. This can include:

- Positive health behaviours like good hygiene and eating well.
- Enjoying hobbies and spending time with loved ones.

|  | 1 (not at all) | 2 (somewhat) | 3 (quite) | 4 (very) | 5 (extremely) |
| --- | --- | --- | --- | --- | --- |
| Important for ageing-in-place |  |  |  |  |  |
| Feasible to routinely measure |  |  |  |  |  |

Comments about indicator *self-care ability*:

[Free text response]

**19. Urinary tract symptoms**

Symptoms that can happen over a long time and might overlap. These can be related to:

- Holding urine (feeling like they really need to go, going often at night, leaking urine)
- Emptying the bladder (weak or on-and-off stream, not emptying the bladder completely)

|  | 1 (not at all) | 2 (somewhat) | 3 (quite) | 4 (very) | 5 (extremely) |
| --- | --- | --- | --- | --- | --- |
| Important for ageing-in-place |  |  |  |  |  |
| Feasible to routinely measure |  |  |  |  |  |

Comments about indicator *urinary tract symptoms*:

[Free text response]

**20. Resilience**

How well a person can handle tough times and bounce back. Indicators could include:

- Being able to cope with problems.
- Recovering well after being sick, hurt, or going through tough times.
- Feeling okay about asking others for help.

|  | 1 (not at all) | 2 (somewhat) | 3 (quite) | 4 (very) | 5 (extremely) |
| --- | --- | --- | --- | --- | --- |
| Important for ageing-in-place |  |  |  |  |  |
| Feasible to routinely measure |  |  |  |  |  |

Comments about indicator *resilience*:

[Free text response]

**21. Adaptability**

This can include:

- How the person anticipates the future.
- Knowing about the care they might need in the future.
- Thinking about and deciding what they want to do in the future.

|  | 1 (not at all) | 2 (somewhat) | 3 (quite) | 4 (very) | 5 (extremely) |
| --- | --- | --- | --- | --- | --- |
| Important for ageing-in-place |  |  |  |  |  |
| Feasible to routinely measure |  |  |  |  |  |

Comments about indicator *adaptability*:

[Free text response]

**22. Self-perceived stress**

How unpredictable, uncontrollable, and overloaded a person finds their life.

|  | 1 (not at all) | 2 (somewhat) | 3 (quite) | 4 (very) | 5 (extremely) |
| --- | --- | --- | --- | --- | --- |
| Important for ageing-in-place |  |  |  |  |  |
| Feasible to routinely measure |  |  |  |  |  |

Comments about indicator *self-perceived stress*:

[Free text response]

**23. Memory and cognitive problems**

These can include issues related to:

- Memory - problems with remembering things, from small details to major memory loss or forgetting everything.
- Attention - trouble concentrating on one thing while blocking out distractions.
- Executive function - difficulties with mental tasks like planning, making decisions, problem-solving, and thinking before acting.

|  | 1 (not at all) | 2 (somewhat) | 3 (quite) | 4 (very) | 5 (extremely) |
| --- | --- | --- | --- | --- | --- |
| Important for ageing-in-place |  |  |  |  |  |
| Feasible to routinely measure |  |  |  |  |  |

Comments about indicator *memory and cognitive problems*:

[Free text response]

**24. Personality factors**

Personality is commonly divided into "five" traits (Goldberg 1993):

- Agreeableness - being friendly and easy to get along with.
- Conscientiousness - being careful, dependable, organised, responsible, and hardworking.
- Extraversion - being outgoing and sociable.
- Neuroticism - being prone to worry, anxiety, and insecurity.
- Openness - being curious and eager to try new things.

|  | 1 (not at all) | 2 (somewhat) | 3 (quite) | 4 (very) | 5 (extremely) |
| --- | --- | --- | --- | --- | --- |
| Important for ageing-in-place |  |  |  |  |  |
| Feasible to routinely measure |  |  |  |  |  |

Comments about indicator *personality factors*:

[Free text response]

**25. Perceived exertion**

- How much effort a person feels their body is putting in.
- Judged by physical feelings during activity, like faster heart rate, heavier breathing, sweating more, and tired muscles.

|  | 1 (not at all) | 2 (somewhat) | 3 (quite) | 4 (very) | 5 (extremely) |
| --- | --- | --- | --- | --- | --- |
| Important for ageing-in-place |  |  |  |  |  |
| Feasible to routinely measure |  |  |  |  |  |

Comments about indicator *perceived exertion*:

[Free text response]

**26. Lifestyle behaviours**

This can include:

- Smoking status
- Alcohol use
- Dietary patterns
- Physical activity levels
- Activity engagement (like housework)
- Oral care
- Regular health check-ups

|  | 1 (not at all) | 2 (somewhat) | 3 (quite) | 4 (very) | 5 (extremely) |
| --- | --- | --- | --- | --- | --- |
| Important for ageing-in-place |  |  |  |  |  |
| Feasible to routinely measure |  |  |  |  |  |

Comments about indicator *lifestyle behaviours*:

[Free text response]

**27. Multimorbidity**

The presence of two or more chronic conditions at the same time, one of which could be either:

- A physical non-communicable disease of long duration (diabetes, heart disease, or cancer).
- A mental health condition of long duration (mood disorder or dementia).
- An infectious disease of long duration (HIV or hepatitis C).

|  | 1 (not at all) | 2 (somewhat) | 3 (quite) | 4 (very) | 5 (extremely) |
| --- | --- | --- | --- | --- | --- |
| Important for ageing-in-place |  |  |  |  |  |
| Feasible to routinely measure |  |  |  |  |  |

Comments about indicator *multimorbidity*:

[Free text response]

**28. Sensory function**

Hearing and vision problems like:

- Hearing difficulties
- Use of a hearing aid
- Loss of vision
- Registered partially or completely blind

|  | 1 (not at all) | 2 (somewhat) | 3 (quite) | 4 (very) | 5 (extremely) |
| --- | --- | --- | --- | --- | --- |
| Important for ageing-in-place |  |  |  |  |  |
| Feasible to routinely measure |  |  |  |  |  |

Comments about indicator *sensory function*:

[Free text response]

**29. Apathy**

Not feeling interested in doing things or being with others. Signs could include:

- Giving up on many hobbies and interests.
- Choosing to stay home instead of going out and trying new things.
- Feeling low on energy.

|  | 1 (not at all) | 2 (somewhat) | 3 (quite) | 4 (very) | 5 (extremely) |
| --- | --- | --- | --- | --- | --- |
| Important for ageing-in-place |  |  |  |  |  |
| Feasible to routinely measure |  |  |  |  |  |

Comments about indicator *apathy*:

[Free text response]

**30. Psychological capacity**

This includes symptoms of depression, anxiety, and sadness like:

- Low satisfaction with life.
- Feeling unhappy most of the time.
- Being in low spirits.
- Feeling helpless, hopeless, and worthless.

|  | 1 (not at all) | 2 (somewhat) | 3 (quite) | 4 (very) | 5 (extremely) |
| --- | --- | --- | --- | --- | --- |
| Important for ageing-in-place |  |  |  |  |  |
| Feasible to routinely measure |  |  |  |  |  |

Comments about indicator *psychological capacity*:

[Free text response]

**31. Foot problems**

Whether the person has swollen feet or reports foot pain.

|  | 1 (not at all) | 2 (somewhat) | 3 (quite) | 4 (very) | 5 (extremely) |
| --- | --- | --- | --- | --- | --- |
| Important for ageing-in-place |  |  |  |  |  |
| Feasible to routinely measure |  |  |  |  |  |

Comments about indicator *foot problems*:

[Free text response]

**32. Polypharmacy**

If a person takes more than 5 prescribed drugs regularly.

|  | 1 (not at all) | 2 (somewhat) | 3 (quite) | 4 (very) | 5 (extremely) |
| --- | --- | --- | --- | --- | --- |
| Important for ageing-in-place |  |  |  |  |  |
| Feasible to routinely measure |  |  |  |  |  |

Comments about indicator *polypharmacy*:

[Free text response]

**33. Dizziness**

- When the person feels they are moving when they are not.
- Feeling disoriented in space.
- Having trouble keeping their balance.

|  | 1 (not at all) | 2 (somewhat) | 3 (quite) | 4 (very) | 5 (extremely) |
| --- | --- | --- | --- | --- | --- |
| Important for ageing-in-place |  |  |  |  |  |
| Feasible to routinely measure |  |  |  |  |  |

Comments about indicator *dizziness*:

[Free text response]

**34. Dyspnoea**

Feelings of breathing discomfort and breathlessness.

|  | 1 (not at all) | 2 (somewhat) | 3 (quite) | 4 (very) | 5 (extremely) |
| --- | --- | --- | --- | --- | --- |
| Important for ageing-in-place |  |  |  |  |  |
| Feasible to routinely measure |  |  |  |  |  |

Comments about indicator *dyspnoea*:

[Free text response]

**35. Sleep-related indicators**

When changes in sleep routine affect daily physical, mental, and emotional functioning. These can include:

- Low sleep duration
- Trouble falling asleep
- Self-perceived sleep quality

|  | 1 (not at all) | 2 (somewhat) | 3 (quite) | 4 (very) | 5 (extremely) |
| --- | --- | --- | --- | --- | --- |
| Important for ageing-in-place |  |  |  |  |  |
| Feasible to routinely measure |  |  |  |  |  |

Comments about indicator s*leep-related indicators*:

[Free text response]

**36. Pressure ulcers**

- Skin and tissue damage when prolonged pressure is exerted.
- Pressure ulcers can happen when people are immobile for long periods.

|  | 1 (not at all) | 2 (somewhat) | 3 (quite) | 4 (very) | 5 (extremely) |
| --- | --- | --- | --- | --- | --- |
| Important for ageing-in-place |  |  |  |  |  |
| Feasible to routinely measure |  |  |  |  |  |

Comments about indicator *pressure ulcers*:

[Free text response]

**37. Meaning in life**

Whether a person finds meaning in their lives, and how much they strive to find meaning and understanding in their lives. This can encompass:

- Purpose - feeling fulfilled or working towards a goal.
- Value - believing that what they do and how they live is good and fair.
- Self-worth - thinking positively about themselves.

|  | 1 (not at all) | 2 (somewhat) | 3 (quite) | 4 (very) | 5 (extremely) |
| --- | --- | --- | --- | --- | --- |
| Important for ageing-in-place |  |  |  |  |  |
| Feasible to routinely measure |  |  |  |  |  |

Comments about indicator *meaning in life*:

[Free text response]

**38. Working capacity**

- How much energy a person puts into their work.
- "Work" can mean any major activity a person does (like paid work, chores, volunteering, studying full-time, taking care of kids, and household tasks).

|  | 1 (not at all) | 2 (somewhat) | 3 (quite) | 4 (very) | 5 (extremely) |
| --- | --- | --- | --- | --- | --- |
| Important for ageing-in-place |  |  |  |  |  |
| Feasible to routinely measure |  |  |  |  |  |

Comments about indicator *working capacity*:

[Free text response]

**39. Perceived autonomy**

- If a person feels like they make their own decisions in life.
- How well they can handle life without needing help from others.
- Being in charge of their own thoughts and feelings without being influenced by others.

|  | 1 (not at all) | 2 (somewhat) | 3 (quite) | 4 (very) | 5 (extremely) |
| --- | --- | --- | --- | --- | --- |
| Important for ageing-in-place |  |  |  |  |  |
| Feasible to routinely measure |  |  |  |  |  |

Comments about indicator *perceived autonomy*:

[Free text response]

**40. Communication problems**

- Speech or language problems.
- Unable to express themselves or their basic needs.
- Uses sign language or technical aid to communicate.

|  | 1 (not at all) | 2 (somewhat) | 3 (quite) | 4 (very) | 5 (extremely) |
| --- | --- | --- | --- | --- | --- |
| Important for ageing-in-place |  |  |  |  |  |
| Feasible to routinely measure |  |  |  |  |  |

Comments about indicator *communication problems*:

[Free text response]

**41. Spiritual health**

Although there is no standard definition, some characteristics include (Whitehead et al. 2021):

- Belief that the spirit or the soul is part of the universal human experience.
- Sense of connectivity to aspects outside the self, for example, with the community, society, family, and nature.
- Relationship with the divine or sacred.
- Beliefs, values, traditions, rituals, and practices.
- Inner strength, peace, and resilience.

|  | 1 (not at all) | 2 (somewhat) | 3 (quite) | 4 (very) | 5 (extremely) |
| --- | --- | --- | --- | --- | --- |
| Important for ageing-in-place |  |  |  |  |  |
| Feasible to routinely measure |  |  |  |  |  |

Comments about indicator *spiritual health*:

[Free text response]

# Section 4: Place

This section includes 18 indicators related to the spatial context inside and around an older person's home, where they interact with the physical, psychological, social, and geographic environment.

We have provided a brief description of each indicator. We ask you to rate each indicator according to two criteria:

Importance: how important is this indicator in understanding how older people with frailty or at risk of frailty are ageing in place?

1 – "not at all important"

2 – "somewhat important"

3 – "quite important"

4 – "very important"

5 – "extremely important"

Feasibility: how feasibly can this indicator be routinely measured in health and social care practice?

1 – "not at all feasible"

2 – "somewhat feasible"

3 – "quite feasible"

4 – "very feasible"

5 – "extremely feasible"

You are also provided with free text boxes below each indicator to add your comments for that indicator. Please refrain from completing the free text boxes with potentially identifiable information such as names, places, etc.

Please note that rating scale responses for all indicators are required, but adding comments is optional. You can return to the survey at any time using the same link, and your responses will be saved.

**1. Life–space mobility**

- Measure of the geographic spaces where a person lives and takes part in roles and activities.
- The geographic spaces include: a person's home, areas outside the home, their neighbourhood, and town or city.

|  | 1 (not at all) | 2 (somewhat) | 3 (quite) | 4 (very) | 5 (extremely) |
| --- | --- | --- | --- | --- | --- |
| Important for ageing-in-place |  |  |  |  |  |
| Feasible to routinely measure |  |  |  |  |  |

Comments about indicator *life–space mobility*:

[Free text response]

**2. Attachment to place**

How emotionally attached a person feels to their home and living environment. Its components could include:

- Place identity - emotional or symbolic attachment formed with a place (home or the neighbourhood).
- Place dependence - how much a place helps a person do the things they want to do and reach their goals.

|  | 1 (not at all) | 2 (somewhat) | 3 (quite) | 4 (very) | 5 (extremely) |
| --- | --- | --- | --- | --- | --- |
| Important for ageing-in-place |  |  |  |  |  |
| Feasible to routinely measure |  |  |  |  |  |

Comments about indicator a*ttachment to place*:

[Free text response]

**3. Land use mix – diversity**

Whether there is a balance of homes and businesses to serve residents in the neighbourhood. One can assess this by the number of:

- Convenience stores, supermarkets, or fruit and vegetable markets
- Banks, post offices, petrol stations, salons
- Leisure facility types
- Medical facilities
- Public parks and/or green spaces
- Community care and welfare centres

|  | 1 (not at all) | 2 (somewhat) | 3 (quite) | 4 (very) | 5 (extremely) |
| --- | --- | --- | --- | --- | --- |
| Important for ageing-in-place |  |  |  |  |  |
| Feasible to routinely measure |  |  |  |  |  |

Comments about indicator *land use mix – diversity*:

[Free text response]

**4. Land use mix – access**

- How easy a person finds accessing facilities.
- These include recreational facilities, stores (like supermarkets and clothing stores), banks, public transport, medical facilities, community and welfare services, and green spaces.

|  | 1 (not at all) | 2 (somewhat) | 3 (quite) | 4 (very) | 5 (extremely) |
| --- | --- | --- | --- | --- | --- |
| Important for ageing-in-place |  |  |  |  |  |
| Feasible to routinely measure |  |  |  |  |  |

Comments about indicator *land use mix – access*:

[Free text response]

**5. Residential density**

- A measure of "urban packing", that is the total number of units of detached, semi-detached, terraced dwellings, and self-contained flats in a buffer area.
- It indicates the amount of activity in a given area that a person can access.

|  | 1 (not at all) | 2 (somewhat) | 3 (quite) | 4 (very) | 5 (extremely) |
| --- | --- | --- | --- | --- | --- |
| Important for ageing-in-place |  |  |  |  |  |
| Feasible to routinely measure |  |  |  |  |  |

Comments about indicator *residential density:*

[Free text response]

**6. Street connectivity**

- The way streets and roads connect homes, shops, parks, and facilities like schools and community centres.
- It includes how the streets are set up, the pavements, how many people walk around, and how easy it is to get from one place to another.

|  | 1 (not at all) | 2 (somewhat) | 3 (quite) | 4 (very) | 5 (extremely) |
| --- | --- | --- | --- | --- | --- |
| Important for ageing-in-place |  |  |  |  |  |
| Feasible to routinely measure |  |  |  |  |  |

Comments about indicator *street connectivity*:

[Free text response]

**7. Access to public transport**

Access to transport systems that help a person get to different places, and whether these systems are disability-friendly and age-friendly.

|  | 1 (not at all) | 2 (somewhat) | 3 (quite) | 4 (very) | 5 (extremely) |
| --- | --- | --- | --- | --- | --- |
| Important for ageing-in-place |  |  |  |  |  |
| Feasible to routinely measure |  |  |  |  |  |

Comments about indicator *access to public transport*:

[Free text response]

**8. Neighbourhood aesthetics**

How attractive and appealing a person finds their living environment. For example:

- If there are nice views or attractive landscaping nearby.
- If there are trees along the streets.
- If there is trash or graffiti around that might make them not want to go out and use the neighbourhood.

|  | 1 (not at all) | 2 (somewhat) | 3 (quite) | 4 (very) | 5 (extremely) |
| --- | --- | --- | --- | --- | --- |
| Important for ageing-in-place |  |  |  |  |  |
| Feasible to routinely measure |  |  |  |  |  |

Comments about indicator *neighbourhood aesthetics:*

[Free text response]

**9. Safety and security in community or neighbourhood**

Feeling safe from things like crime and accidents in the neighbourhood, and feeling relaxed in their surroundings. Indicators can include:

- How much crime happens in the area.
- Whether the streets are well lit at night.
- If a person feels safe walking around after dark.

|  | 1 (not at all) | 2 (somewhat) | 3 (quite) | 4 (very) | 5 (extremely) |
| --- | --- | --- | --- | --- | --- |
| Important for ageing-in-place |  |  |  |  |  |
| Feasible to routinely measure |  |  |  |  |  |

Comments about indicator *safety and security in community or neighbourhood*:

[Free text response]

**10. Residential and neighbourhood satisfaction**

How a person views their housing and neighbourhood environments. For example:

- Whether they think their home is good enough for them.
- What they think about aspects like schools, doctors, parks, and how they get around.
- How they get along with their neighbours.

|  | 1 (not at all) | 2 (somewhat) | 3 (quite) | 4 (very) | 5 (extremely) |
| --- | --- | --- | --- | --- | --- |
| Important for ageing-in-place |  |  |  |  |  |
| Feasible to routinely measure |  |  |  |  |  |

Comments about indicator *residential and neighbourhood satisfaction*:

[Free text response]

**11. Neighbourhood walkability**

How neighbourhoods and their designs affect physical activity. Some indicators include:

- If it is easy to walk around.
- How comfortable it is (like if the streets are wide and smooth, and if there are places to take a break).
- How easy it is to get around and find things (like if there are signs and facilities are easy to reach).

|  | 1 (not at all) | 2 (somewhat) | 3 (quite) | 4 (very) | 5 (extremely) |
| --- | --- | --- | --- | --- | --- |
| Important for ageing-in-place |  |  |  |  |  |
| Feasible to routinely measure |  |  |  |  |  |

Comments about indicator *neighbourhood walkability:*

[Free text response]

**12. Neighbourhood resources**

Whether a person can access places for social interaction or intellectual stimulation like:

- Recreational centres (like fitness centres, swimming pools)
- Institutions like schools, places of worship, and libraries
- Community centres and volunteering organisations

|  | 1 (not at all) | 2 (somewhat) | 3 (quite) | 4 (very) | 5 (extremely) |
| --- | --- | --- | --- | --- | --- |
| Important for ageing-in-place |  |  |  |  |  |
| Feasible to routinely measure |  |  |  |  |  |

Comments about indicator *neighbourhood resources*:

[Free text response]

**13. Neighbourhood disadvantage**

A neighbourhood may be disadvantaged based on indicators like:

- Income
- Education and skills training
- Employment
- Barriers to housing and services
- Health deprivation and disability
- Crime and living environment

In the UK, we use the Index of Multiple Deprivation (IMD), which measures relative deprivation.

|  | 1 (not at all) | 2 (somewhat) | 3 (quite) | 4 (very) | 5 (extremely) |
| --- | --- | --- | --- | --- | --- |
| Important for ageing-in-place |  |  |  |  |  |
| Feasible to routinely measure |  |  |  |  |  |

Comments about indicator *neighbourhood disadvantage:*

[Free text response]

**14. Home environment – social dimension**

This could include:

- Who lives in the house with the person.
- How happy the person is with the people they live with or who come to visit.
- If they feel comfortable inviting friends or family over to their home.

|  | 1 (not at all) | 2 (somewhat) | 3 (quite) | 4 (very) | 5 (extremely) |
| --- | --- | --- | --- | --- | --- |
| Important for ageing-in-place |  |  |  |  |  |
| Feasible to routinely measure |  |  |  |  |  |

Comments about indicator *home environment – social dimension*:

[Free text response]

**15. Home environment – occupational dimension**

This could include:

- Whether the home is easy to clean.
- Whether people can easily move around in their home.
- Whether people can easily do the activities they need to (e.g. shower, toilet use) and enjoy (e.g. leisure activities) in their home.

|  | 1 (not at all) | 2 (somewhat) | 3 (quite) | 4 (very) | 5 (extremely) |
| --- | --- | --- | --- | --- | --- |
| Important for ageing-in-place |  |  |  |  |  |
| Feasible to routinely measure |  |  |  |  |  |

Comments about indicator *home environment – occupational dimension:*

[Free text response]

**16. Home environment – personal dimension**

This could include:

- How much a person feels like their home is private and safe.
- If they are happy with how their home looks inside and where it is located.
- How emotionally connected they feel to their home.

|  | 1 (not at all) | 2 (somewhat) | 3 (quite) | 4 (very) | 5 (extremely) |
| --- | --- | --- | --- | --- | --- |
| Important for ageing-in-place |  |  |  |  |  |
| Feasible to routinely measure |  |  |  |  |  |

Comments about indicator *home environment – personal dimension:*

[Free text response]

**17. Home environment – physical dimension (structure, services, and facilities)**

Indicators could include:

- How many floors the house has.
- Barriers outside, at the doors, and inside the home that might make it hard to move around.
- Any major problems with how the house is built.
- Presence of fall risks (rugs, wires or cords, uneven stairs, lack of proper lighting, etc.).

|  | 1 (not at all) | 2 (somewhat) | 3 (quite) | 4 (very) | 5 (extremely) |
| --- | --- | --- | --- | --- | --- |
| Important for ageing-in-place |  |  |  |  |  |
| Feasible to routinely measure |  |  |  |  |  |

Comments about indicator *home environment – physical dimension (structure, services, and facilities):*

[Free text response]

**18. Home environment – physical dimension (ambience and space)**

Indicators could include:

- Satisfaction with ambience of the home (e.g. a view, breeze, or sunshine).
- How warm or cool the person feels inside their home.
- If they feel like they have enough space in the home.
- Lighting and brightness in the home.

|  | 1 (not at all) | 2 (somewhat) | 3 (quite) | 4 (very) | 5 (extremely) |
| --- | --- | --- | --- | --- | --- |
| Important for ageing-in-place |  |  |  |  |  |
| Feasible to routinely measure |  |  |  |  |  |

Comments about indicator *home environment – physical dimension (ambience and space):*

[Free text response]

# Section 5: Social networks

This section of the survey includes eight indicators related to the frequency and nature of social interactions that older people have.

We have provided a brief description of each indicator. We ask you to rate each indicator according to two criteria:

Importance: how important is this indicator in understanding how older people with frailty or at risk of frailty are ageing in place?

1 – "not at all important"

2 – "somewhat important"

3 – "quite important"

4 – "very important"

5 – "extremely important"

Feasibility: how feasibly can this indicator be routinely measured in health and social care practice?

1 – "not at all feasible"

2 – "somewhat feasible"

3 – "quite feasible"

4 – "very feasible"

5 – "extremely feasible"

You are also provided with free text boxes below each indicator to add your comments for that indicator. Please refrain from completing the free text boxes with potentially identifiable information such as names, places, etc.

Please note that rating scale responses for all indicators are required, but adding comments is optional. You can return to the survey at any time using the same link, and your responses will be saved.

**1. Social participation – engagement**

A person's involvement in activities that lets them interact with others in their community, like:

- Going to religious or cultural events.
- Doing leisure activities with friends or family.
- Helping out in their community or getting involved in civic activities.
- Going out with friends or family, or having them over to their home.

|  | 1 (not at all) | 2 (somewhat) | 3 (quite) | 4 (very) | 5 (extremely) |
| --- | --- | --- | --- | --- | --- |
| Important for ageing-in-place |  |  |  |  |  |
| Feasible to routinely measure |  |  |  |  |  |

Comments about indicator *social participation – engagement*:

[Free text response]

**2. Social participation – access**

Whether a person has opportunities to socialise and interact with others in their community, like:

- Having family or friends they spend time with.
- Being able to go to places for recreational activities, like sports centres or community centres.
- Being able to go to parks or green areas near their home.

|  | 1 (not at all) | 2 (somewhat) | 3 (quite) | 4 (very) | 5 (extremely) |
| --- | --- | --- | --- | --- | --- |
| Important for ageing-in-place |  |  |  |  |  |
| Feasible to routinely measure |  |  |  |  |  |

Comments about indicator *social participation – access*:

[Free text response]

**3. Social participation – awareness**

Whether a person knows about opportunities to socialise and interact with others in their area, like:

- Activities happening at places like sports centres or community centres.
- Volunteering activities.
- Hobby groups like book clubs, walking groups, etc.

|  | 1 (not at all) | 2 (somewhat) | 3 (quite) | 4 (very) | 5 (extremely) |
| --- | --- | --- | --- | --- | --- |
| Important for ageing-in-place |  |  |  |  |  |
| Feasible to routinely measure |  |  |  |  |  |

Comments about indicator *social participation – awareness*:

[Free text response]

**4. Social participation – satisfaction**

Whether a person is happy with the social activities they do, and if they are doing as much as they want. This could include:

- How they feel about the quality of the social activities they can do.
- If they have any problems socialising with others.
- How happy they are with how they interact with places, groups, and organisations in their community.

|  | 1 (not at all) | 2 (somewhat) | 3 (quite) | 4 (very) | 5 (extremely) |
| --- | --- | --- | --- | --- | --- |
| Important for ageing-in-place |  |  |  |  |  |
| Feasible to routinely measure |  |  |  |  |  |

Comments about indicator *social participation – satisfaction*:

[Free text response]

**5. Social capital**

A type of resource found in both casual interactions with others and involvement in formal groups. It has two parts:

1. Cognitive social capital:

- How much people in the community are seen as helpful.
- When community members share valuable things to help each other out.

2. Structural social capital:

- Being part of any organised group in the community.
- Working together with others in the community to fix a problem.

|  | 1 (not at all) | 2 (somewhat) | 3 (quite) | 4 (very) | 5 (extremely) |
| --- | --- | --- | --- | --- | --- |
| Important for ageing-in-place |  |  |  |  |  |
| Feasible to routinely measure |  |  |  |  |  |

Comments about indicator *social capital:*

[Free text response]

**6. Social cohesion**

How close people are in the community and how much they help each other out. Indicators could include:

- How much people think their friendships and connections with others mean.
- If people are ready to team up with others to make the neighbourhood better.
- If neighbours and community members know each other well.

|  | 1 (not at all) | 2 (somewhat) | 3 (quite) | 4 (very) | 5 (extremely) |
| --- | --- | --- | --- | --- | --- |
| Important for ageing-in-place |  |  |  |  |  |
| Feasible to routinely measure |  |  |  |  |  |

Comments about indicator *social cohesion:*

[Free text response]

**7. Social networks**

The nature of relationships people have with children, grandchildren, siblings, relatives, friends, and others in the neighbourhood. Indicators could include:

- Where and who they live with.
- If they have family or friends they spend time with or talk to.
- If they have family or friends they speak with about personal matters.
- How often they go out with family or friends or visit each other's homes.

|  | 1 (not at all) | 2 (somewhat) | 3 (quite) | 4 (very) | 5 (extremely) |
| --- | --- | --- | --- | --- | --- |
| Important for ageing-in-place |  |  |  |  |  |
| Feasible to routinely measure |  |  |  |  |  |

Comments about indicator *social networks*:

[Free text response]

**8. Satisfaction with social relationships**

How people feel about their connections with family, friends, and people in the neighbourhood. Indicators could include:

- If they feel helpful to others and the community.
- If they are happy with how much time they spend with family and friends.
- If they are happy with their involvement in social and family activities.

|  | 1 (not at all) | 2 (somewhat) | 3 (quite) | 4 (very) | 5 (extremely) |
| --- | --- | --- | --- | --- | --- |
| Important for ageing-in-place |  |  |  |  |  |
| Feasible to routinely measure |  |  |  |  |  |

Comments about indicator *satisfaction with social relationships*:

[Free text response]

# Section 6: Support

This section of the survey includes eight indicators related to two kinds of support available to older people: formal support (provided by professionals and service providers) and informal support (provided by friends, family, neighbours, etc.).

We have provided a brief description of each indicator. We ask you to rate each indicator according to two criteria:

Importance: how important is this indicator in understanding how older people with frailty or at risk of frailty are ageing in place?

1 – "not at all important"

2 – "somewhat important"

3 – "quite important"

4 – "very important"

5 – "extremely important"

Feasibility: how feasibly can this indicator be routinely measured in health and social care practice?

1 – "not at all feasible"

2 – "somewhat feasible"

3 – "quite feasible"

4 – "very feasible"

5 – "extremely feasible"

You are also provided with free text boxes below each indicator to add your comments for that indicator. Please refrain from completing the free text boxes with potentially identifiable information such as names, places, etc.

Please note that rating scale responses for all indicators are required, but adding comments is optional. You can return to the survey at any time using the same link, and your responses will be saved.

**1. Food security**

Whether people have access to enough good-quality food at all times for an active and healthy lifestyle. This could include:

- Quality and types of food and drinks that people can buy and consume.
- Financial capability to purchase food and drinks.
- Food store availability near one's home.
- Whether support is needed to buy food and prepare meals.

|  | 1 (not at all) | 2 (somewhat) | 3 (quite) | 4 (very) | 5 (extremely) |
| --- | --- | --- | --- | --- | --- |
| Important for ageing-in-place |  |  |  |  |  |
| Feasible to routinely measure |  |  |  |  |  |

Comments about indicator *food security*:

[Free text response]

**2. Access to social support**

The support accessible to a person through social ties to other individuals, groups, and the larger community. Indicators could include:

- Whether there is someone around when the person is in need.
- Whether the person gets emotional help and support from friends and/or family.
- Whether a friend or family member is willing to help them make decisions.

|  | 1 (not at all) | 2 (somewhat) | 3 (quite) | 4 (very) | 5 (extremely) |
| --- | --- | --- | --- | --- | --- |
| Important for ageing-in-place |  |  |  |  |  |
| Feasible to routinely measure |  |  |  |  |  |

Comments about indicator a*ccess to social support*:

[Free text response]

**3. Satisfaction with social support**

How happy people are with the help they give and receive from family and friends. Indicators could include:

- If family and friends understand them.
- If they feel helpful to their family and friends.
- If they feel like they have a clear role in their family and with their friends.

|  | 1 (not at all) | 2 (somewhat) | 3 (quite) | 4 (very) | 5 (extremely) |
| --- | --- | --- | --- | --- | --- |
| Important for ageing-in-place |  |  |  |  |  |
| Feasible to routinely measure |  |  |  |  |  |

Comments about indicator *satisfaction with social support*:

[Free text response]

**4. Ability to provide social support**

A person's capacity to provide social support to family and friends. Indicators could include:

- Whether there is someone who shares their complaints or worries with the person (providing emotional support).
- Ability to take care of someone if they were ill or in bed for several days (providing instrumental support).

|  | 1 (not at all) | 2 (somewhat) | 3 (quite) | 4 (very) | 5 (extremely) |
| --- | --- | --- | --- | --- | --- |
| Important for ageing-in-place |  |  |  |  |  |
| Feasible to routinely measure |  |  |  |  |  |

Comments about indicator *ability to provide social support*:

[Free text response]

**5. Social services and formal care – awareness**

Whether the person knows about social services and formal care support available in their community. Such support can include:

- Financial and legal services
- Benefits counselling
- Employment placement	Homecare services
- Long-term care services

|  | 1 (not at all) | 2 (somewhat) | 3 (quite) | 4 (very) | 5 (extremely) |
| --- | --- | --- | --- | --- | --- |
| Important for ageing-in-place |  |  |  |  |  |
| Feasible to routinely measure |  |  |  |  |  |

Comments about indicator *social services and formal care – awareness:*

[Free text response]

**6. Social services and formal care – access**

- Whether the person feels confident and comfortable about getting help from social services and care support in their community.
- This also includes getting information and advice about these services or benefits.

|  | 1 (not at all) | 2 (somewhat) | 3 (quite) | 4 (very) | 5 (extremely) |
| --- | --- | --- | --- | --- | --- |
| Important for ageing-in-place |  |  |  |  |  |
| Feasible to routinely measure |  |  |  |  |  |

Comments about indicator *social services and formal care – access:*

[Free text response]

**7. Social services and formal care – satisfaction**

- How a person sees the formal care and services available to them.
- How they view the quality and completeness of the care they get or what they expect to get if they need these services.

|  | 1 (not at all) | 2 (somewhat) | 3 (quite) | 4 (very) | 5 (extremely) |
| --- | --- | --- | --- | --- | --- |
| Important for ageing-in-place |  |  |  |  |  |
| Feasible to routinely measure |  |  |  |  |  |

Comments about indicator *social services and formal care – satisfaction*:

[Free text response]

**8. Pet ownership**

Whether the person currently lives with a pet (such as a cat, dog, birds, fish, etc.) in their home.

|  | 1 (not at all) | 2 (somewhat) | 3 (quite) | 4 (very) | 5 (extremely) |
| --- | --- | --- | --- | --- | --- |
| Important for ageing-in-place |  |  |  |  |  |
| Feasible to routinely measure |  |  |  |  |  |

Comments about indicator *pet ownership*:

[Free text response]

# Section 7: Technology

This section of the survey includes 14 indicators related to how older people perceive and use technology. The types of technology include those used for mobility support, information and communication technology (ICT), biotechnology, and ambient intelligence.

We have provided a brief description of each indicator. We ask you to rate each indicator according to two criteria:

Importance: how important is this indicator in understanding how older people with frailty or at risk of frailty are ageing in place?

1 – "not at all important"

2 – "somewhat important"

3 – "quite important"

4 – "very important"

5 – "extremely important"

Feasibility: how feasibly can this indicator be routinely measured in health and social care practice?

1 – "not at all feasible"

2 – "somewhat feasible"

3 – "quite feasible"

4 – "very feasible"

5 – "extremely feasible"

You are also provided with free text boxes below each indicator to add your comments for that indicator. Please refrain from completing the free text boxes with potentially identifiable information such as names, places, etc.

Please note that rating scale responses for all indicators are required, but adding comments is optional. You can return to the survey at any time using the same link, and your responses will be saved.

**1. General satisfaction with technology**

Whether users feel like the technology matches up with what they need it to do.

|  | 1 (not at all) | 2 (somewhat) | 3 (quite) | 4 (very) | 5 (extremely) |
| --- | --- | --- | --- | --- | --- |
| Important for ageing-in-place |  |  |  |  |  |
| Feasible to routinely measure |  |  |  |  |  |

Comments about indicator *general satisfaction with technology:*

[Free text response]

**2. Technology ownership**

Whether a person owns

- ICT devices like computers, tablets, mobile phones, smartphones, or activity-tracking watches.
- Smart home devices like motion sensor lights, home security systems, voice-activated assistants, emergency alert systems, etc.

|  | 1 (not at all) | 2 (somewhat) | 3 (quite) | 4 (very) | 5 (extremely) |
| --- | --- | --- | --- | --- | --- |
| Important for ageing-in-place |  |  |  |  |  |
| Feasible to routinely measure |  |  |  |  |  |

Comments about indicator *technology ownership*:

[Free text response]

**3. Internet access**

Whether a person has internet access at home or can easily get online at places like libraries or community centres.

|  | 1 (not at all) | 2 (somewhat) | 3 (quite) | 4 (very) | 5 (extremely) |
| --- | --- | --- | --- | --- | --- |
| Important for ageing-in-place |  |  |  |  |  |
| Feasible to routinely measure |  |  |  |  |  |

Comments about indicator *internet access*:

[Free text response]

**4. Internet use**

Whether a person uses the internet for purposes like communication, general browsing, social media, entertainment, transactions, etc.

|  | 1 (not at all) | 2 (somewhat) | 3 (quite) | 4 (very) | 5 (extremely) |
| --- | --- | --- | --- | --- | --- |
| Important for ageing-in-place |  |  |  |  |  |
| Feasible to routinely measure |  |  |  |  |  |

Comments about indicator *internet use:*

[Free text response]

**5. Perceived usefulness of technology**

The belief that using the technology will make the user's tasks or roles easier and better in terms of getting things done, like daily activities.

|  | 1 (not at all) | 2 (somewhat) | 3 (quite) | 4 (very) | 5 (extremely) |
| --- | --- | --- | --- | --- | --- |
| Important for ageing-in-place |  |  |  |  |  |
| Feasible to routinely measure |  |  |  |  |  |

Comments about indicator *perceived usefulness of technology:*

[Free text response]

**6. Perceived ease of technology use**

How much a user thinks using a technology will not need much physical or mental effort.

|  | 1 (not at all) | 2 (somewhat) | 3 (quite) | 4 (very) | 5 (extremely) |
| --- | --- | --- | --- | --- | --- |
| Important for ageing-in-place |  |  |  |  |  |
| Feasible to routinely measure |  |  |  |  |  |

Comments about indicator *perceived ease of technology use*:

[Free text response]

**7. Attitude towards technology use**

Positive or negative feelings about using a technology for managing health or staying connected to people. Indicators could include:

- Worries about privacy.
- If the user plans to keep using the technology in the future.

|  | 1 (not at all) | 2 (somewhat) | 3 (quite) | 4 (very) | 5 (extremely) |
| --- | --- | --- | --- | --- | --- |
| Important for ageing-in-place |  |  |  |  |  |
| Feasible to routinely measure |  |  |  |  |  |

Comments about indicator *attitude towards technology use*:

[Free text response]

**8. Intent to use technology**

How much a person has actively decided to use the technology both now and in the future.

|  | 1 (not at all) | 2 (somewhat) | 3 (quite) | 4 (very) | 5 (extremely) |
| --- | --- | --- | --- | --- | --- |
| Important for ageing-in-place |  |  |  |  |  |
| Feasible to routinely measure |  |  |  |  |  |

Comments about indicator *intent to use technology:*

[Free text response]

**9. Reliability of the technology**

Whether users and providers think the technology is safe, secure, effective, and satisfactory.

|  | 1 (not at all) | 2 (somewhat) | 3 (quite) | 4 (very) | 5 (extremely) |
| --- | --- | --- | --- | --- | --- |
| Important for ageing-in-place |  |  |  |  |  |
| Feasible to routinely measure |  |  |  |  |  |

Comments about indicator *reliability of the technology:*

[Free text response]

**10. Comfort with technology use**

- How comfortable or nervous the person feels about using technology in general.
- If the person feels happy or worried when using technology.
- Whether users have had good experiences with using technology overall.

|  | 1 (not at all) | 2 (somewhat) | 3 (quite) | 4 (very) | 5 (extremely) |
| --- | --- | --- | --- | --- | --- |
| Important for ageing-in-place |  |  |  |  |  |
| Feasible to routinely measure |  |  |  |  |  |

Comments about indicator *comfort with technology use*:

[Free text response]

**11. Reasons for internet and technology use**

These could include:

- Staying connected with friends and family
- Participating in social activities
- Performing daily tasks
- Managing health
- Leisure (like internet shopping) and banking

|  | 1 (not at all) | 2 (somewhat) | 3 (quite) | 4 (very) | 5 (extremely) |
| --- | --- | --- | --- | --- | --- |
| Important for ageing-in-place |  |  |  |  |  |
| Feasible to routinely measure |  |  |  |  |  |

Comments about indicator *reasons for internet and technology use:*

[Free text response]

**12. Digital literacy**

The skills needed to live, learn, work, participate, and do well in a digital world. These could include:

- Knowing how to use digital tools, like computers or phones, to find, judge, use, and make information.
- Being able to understand and use information in different ways when it is shown on digital devices.
- Being able to do things well in a digital setting.

|  | 1 (not at all) | 2 (somewhat) | 3 (quite) | 4 (very) | 5 (extremely) |
| --- | --- | --- | --- | --- | --- |
| Important for ageing-in-place |  |  |  |  |  |
| Feasible to routinely measure |  |  |  |  |  |

Comments about indicator *digital literacy:*

[Free text response]

**13. Subjective norms**

How a user's digital habits are affected by what their social circle thinks (like family). For example, if family members suggest or warn against using specific technology.

|  | 1 (not at all) | 2 (somewhat) | 3 (quite) | 4 (very) | 5 (extremely) |
| --- | --- | --- | --- | --- | --- |
| Important for ageing-in-place |  |  |  |  |  |
| Feasible to routinely measure |  |  |  |  |  |

Comments about indicator *subjective norms:*

[Free text response]

**14. Physical environment**

Whether a person's home or living space make it easy to use technology or set up devices like motion-sensing lights and security systems.

|  | 1 (not at all) | 2 (somewhat) | 3 (quite) | 4 (very) | 5 (extremely) |
| --- | --- | --- | --- | --- | --- |
| Important for ageing-in-place |  |  |  |  |  |
| Feasible to routinely measure |  |  |  |  |  |

Comments about indicator *physical environment*:

[Free text response]
