# Supplementary material for: Proxy indicators to support independent ageing-in-place in older people with frailty: a Delphi-style expert consultation survey
Source: BMC Geriatr. 2026 Jul 28;26:1335. doi: 10.1186/s12877-026-07968-9 (PMC13693169; doi:10.1186/s12877-026-07968-9)
Supplement: Supplementary file 4 [file 12877_2026_7968_MOESM5_ESM.docx]

Supplement 5

# Thematic summary of the discussions with public contributors regarding their feedback and insights on the ageing-in-place indicators.

| Questions / Themes | Summary |
| --- | --- |
| Important indicators (explicitly specified by the public contributors) | - Access to public transport - Digital literacy - Fatigue - Independence and confidence in activities of daily living (ADLs) - Land use mix – access - Loneliness - Multimorbidity - Neighbourhood resources - Physical performance and mobility - Sensory function - Sleep-related indicators - Social networks - Social participation – access - Social support-related indicators - Social vulnerability |
| Important indicators (that emerged implicitly during the discussions) | - Ability to provide support - Adaptability - Apathy - Attachment to place (adapting home for later life) - Falls-related indicators - Independence and confidence in ADLs - Meaning in life - Memory and cognition - Neighbourhood walkability - Perceived autonomy - Residential and neighbourhood satisfaction - Resilience - Satisfaction with social support and relationships - Self-care ability - Social cohesion - Social services and formal care – satisfaction - Street connectivity - Technology-related indicators (perceived ease of use, attitudes, reasons for use, intent to use, perceived usefulness, comfort with using technology, subjective norms, reliability) - Working capacity |
| Perceptions about routine assessment of indicators | - Mixed views on who is in the best position to assess these indicators (older people themselves, carers / family members, or health and social care professionals). - Clinical indicators may be best assessed by health and social care professionals. - Indicators related to cognitive losses, withdrawal and loss of interest, ability to think rationally, and physical issues may be best captured by carers / family members. - Formal health and social care professionals spend limited time with patients, so they are unable to routinely assess and record information related to older people’s environments and social situations. - Lack of integration and centralisation of patients’ medical information and care records among different health and social care services. |
| Perceptions around technology-related indicators | - Consensus on the importance of technology-related indicators for older people ageing in place. - Variations in technology ownership and use among older people. - “Digital literacy” was emphasised, given digital shift in services such as healthcare and banking. - Educating older people on using technology, why it is needed to enable them to age in place, and digital services is recommended. - Technology is beneficial to keep in touch with loved ones, book appointments online, and online shopping, especially for carers. However, there were concerns around technology related to smart home modifications. - With increasing frailty, technology may become more burdensome and frustrating to use, which may increase the risk of dependency to perform digital-related tasks. |
| Future directions for research and practice | - Routine collection of social and environmental, i.e. non-clinical information. - Involving volunteers (such as those involved in befriending services) to collect information on the environmental and social situations of older people. - Integration of clinical and non-clinical information that can be accessed by all service providers to facilitate tailored care delivery. - Exploring hospital-at-home and better homecare services to promote ageing-in-place. - Qualitative research involving older people and carers about ageing-in-place indicators. - Educating older people about technology and providing digital support to bridge the digital divide in older people. - Addressing social indicators such as loneliness. |
